# Modelling effects of time-variable exposure to the pyrethroid beta-cyfluthrin on rainbow trout early life stages

**DOI:** 10.1186/s12302-018-0162-0

**Published:** 2018-09-18

**Authors:** Elke I. Zimmer, Thomas G. Preuss, Steve Norman, Barbara Minten, Virginie Ducrot

**Affiliations:** 1 ibacon GmbH, Arheilger Weg 17, 64380 Roßdorf, Germany; 2 Bayer AG CropScience Division, 40789 Monheim Am Rhein, Germany; 3 RidgewayEco Harwell Innovation Centre, Oxfordshire, OX11 0QG UK; 4 ADAMA Deutschland GmbH, Edmund-Rumpler-Str. 6, 51149 Cologne, Germany

**Keywords:** DEB, TKTD, Pyrethroid, Mechanistic

## Abstract

**Background:**

Available literature and regulatory studies show that the severity of effects of beta-cyfluthrin (a synthetic pyrethroid) on fish is influenced by the magnitude and duration of exposure. To investigate how the exposure pattern to beta-cyfluthrin (constant vs peak) may influence the response of the fish, we used a mechanistic effect model to predict the survival and growth of the rainbow trout over its early life stages (i.e. egg, alevin and swim-up fry). We parameterized a toxicokinetic–toxicodynamic (TKTD) module in combination with a dynamic energy budget model enabling us to describe uptake and elimination, as well as to predict the threshold concentration for survival and sublethal effects (feeding behaviour and growth). This effect model was calibrated using data from an early life stage experiment where trout was exposed to a constant concentration of cyfluthrin. The model was validated by comparing model predictions to independent data from a pulsed-exposure study with early life stages of rainbow trout.

**Results:**

The co-occurrence of effects on behaviour and growth raised the possibility that these were interrelated, i.e. impairment of feeding behaviour may have led to reduced food intake and slower growth. We, therefore, included ‘effect on feeding’ as mode of action in the TKTD module. At higher concentrations, the constant exposure led to death. The model was able to adequately capture this effect pattern in the calibration. The model was able to adequately predict the response of fish eggs, alevins and swim-up fry, from both the qualitative (response pattern) and quantitative points of view.

**Conclusions:**

Since the model was successfully validated, it can be used to predict survival and growth of early life stages under various realistic time-variable exposure profiles (e.g. profiles from FOCUS surface water modelling) of beta-cyfluthrin.

**Electronic supplementary material:**

The online version of this article (10.1186/s12302-018-0162-0) contains supplementary material, which is available to authorized users.

## Background

Toxicokinetic–toxicodynamic (TKTD) models have been discussed as potential tools to provide an understanding of effects of compounds on the physiology of organisms and their life-history traits, and most importantly, to capture these effects as a function of time [1]. In recent years, the European Commission and the European Food Safety Authority (EFSA) have recognized the relevance of these tools in the context of regulatory risk assessment of plant protection products [2]. In recent guidance documents and scientific opinions related to risk assessment for non-target organisms (e.g. birds, mammals, aquatic animals and plants, soil organisms, bees and other non-target arthropods) mechanistic effect models have been mentioned as refinement options for the ecotoxicological risk assessment of Plant Protection Products (see e.g. [3] for aquatic organisms). In these EFSA documents, models are suggested as means for improving the understanding of the driving mechanisms of effect and recovery patterns, to extrapolate to different exposure profiles, seasons, geographic zones and species, as well as to link sublethal endpoints from standard ecotoxicological studies (e.g. growth and reproduction) to the specific protection goals (e.g. population sustainability). Recently, an EFSA specialist panel published an opinion paper that provides further guidance on how to use TKTD models within the regulatory risk assessment framework [4].

Among available TKTD models, dynamic energy budget (DEB) models seem particularly suited to deal with organism-level effects such as survival, growth and reproduction simultaneously [1, 5]. In a DEB model, the basic metabolic processes such as feeding, growth, reproduction, maintenance and ageing are included in a single modelling framework. The same model structure can in general be used for various organisms. Species differences are mainly reflected in the parameter values defining the energetic fluxes between metabolic processes [6]. However, some details concerning early life stages, reproductive strategies, and starvation responses are species specific. Therefore, a DEB model can be viewed as a modular tool with a core part that describes the general physiology of an animal and a suite of extensions (or modules) that describe the species-specific features. Modules needed depend on the species and particular risk assessment question at hand. Generally, these types of models belong to the family of bioenergetics models, which have been suggested for the refinement of Tier-2 in the guidance document for Good Modelling Practice [2]. Both OECD and EFSA have shown interest in possibilities of using DEB models in ecotoxicology. Biological endpoints derived from DEBtox models (i.e. DEB models with an integrated module for effects of toxicants [7, 8] were deemed adequate tools in the OECD guidance document on statistical approaches for the analysis of ecotoxicity data [9, 10]. More recently, some of the EFSA tenders have explicitly stated DEBtox as a tool that should be included in the environmental risk assessment (e.g. call for mixture toxicity in bees, OC/EFSA/SCER/2013/02; population dynamics of aquatic and terrestrial organisms using DEB models, OC/EFSA/SCER/2015/01). The newly published EFSA opinion paper on TKTD models [4] recognises the great potential of the DEBtox modelling approach for future use in prospective ERA for pesticides. However, it is currently seen as limited to research applications due to a lack of well-documented applications in this field.

The aim of this study was to test the suitability of a TKTD model based on DEB theory to explain and predict the effects of (beta-)cyfluthrin on early life stages of rainbow trout (*Oncorhynchus mykiss*). This fish is the only cold water fish that is recommended for ecotoxicological testing by the OECD [11], and it is the standard test species for acute tests based on data requirements. For chronic tests, only an ELS is possible with trouts and then often other species are tested. It is of great economical importance in many regions of the world and has been identified as one of the most sensitive species to (beta-)cyfluthrin based on acute toxicity data [12].

Beta-cyfluthrin (and the racemic mixture, cyfluthrin) is a synthetic pyrethroid insecticide. Cyfluthrin is a mixture of four isomers (two* cis* and two* trans*) while beta-cyfluthrin only contains the two active isomers (one *cis* and one *trans*) [13]. Since the two isomers that are left out are not active, we assume that the ecotoxicological effect of beta-cyfluthrin and cyfluthrin is the same. This group of chemicals has a neurological mode of action in insects (e.g. [14]). The effects of cyfluthrin on rainbow trout were evaluated in 1985 in an early life stage (ELS) study using constant exposure, conducted for regulatory purposes (Bayer AG, unpublished; hereafter referred to as ‘Experiment 1’). In this study, substantial mortality was observed in mean measured (mm) concentrations of 31.8 ng/L and higher, and significant effects on growth were observed in concentrations of 17.7 ng/L (mm) and higher. In later publications it has been concluded that pyrethroids in general tend to be neurotoxic to fish [15] and that they have been found to induce locomotory abnormalities in rainbow trout [16]. Using the adverse-outcome pathway (AOP) concept, Groh et al. [17] highlighted that locomotion impairment by pyrethroids results in reduction in food intake and consequential reduced growth. Nevertheless, feeding behaviour of fish—as an assessed parameter—had not yet been monitored in a beta-cyfluthrin or cyfluthrin study.

In 2016, an ELS study with beta-cyfluthrin using a realistic worst case time-variable (peak) exposure profile was conducted for regulatory purposes (ADAMA, unpublished; hereafter referred to as ‘Experiment 2’). In this study, three different life stages were tested: ‘Cohort C’ (exposed as eggs), ‘Cohort B’ (exposed as alevins) and ‘Cohort A’ (exposed as swim-up fry). Feeding behaviour was included as a new explanatory endpoint in addition to growth, clinical signs and mortality. Cohorts were exposed to two static dosing events with a 14-day natural dissipation interval under the presence of sediment. Nominal peak-exposure concentrations were 0 (control), 32, 48, 72, 180 and 450 ng/L. No effects on survival were observed. The pulsed exposure consisted of two static dosing events with natural dissipation in the presence of sediment. A statistically significant impairment of feeding behaviour was observed at 48, 72, 180 and 450 ng/L (nominal). The severity of impairment was concentration dependent. At 48 and 72 ng/L, the fry returned to normal feeding within 96 h of the exposure peak. A statistically significant difference in growth was found for swim-up fry exposed to a peak of 72, 180 and 450 ng/L (3.4% shorter body length than the control in the highest concentration). Based on observations made in Experiment 2 and the conclusions put forward by Groh et al., we hypothesize that observed clinical signs impaired feeding behaviour and consequently growth in both experiments.

## Results

### Performance of the physiological model

The available DEB model for rainbow trout was tested for suitability to be used in this study (see "Methods" section). All data except the data on oxygen consumption met our performance criteria, meaning that the model output for the study and the empirical results did not differ more than the previously defined performance criteria of 15% deviation (see Additional file 1: Table S2). Since oxygen consumption is irrelevant for the present study, we concluded that the DEB model and the estimated parameters were fit for our purpose of predicting survival, length and bodyweight of rainbow trout fry in ELS studies.

### Calibration of the DEB model (including the TKTD module): model predictions compared with data from Experiment 1

**Fig. 1:**
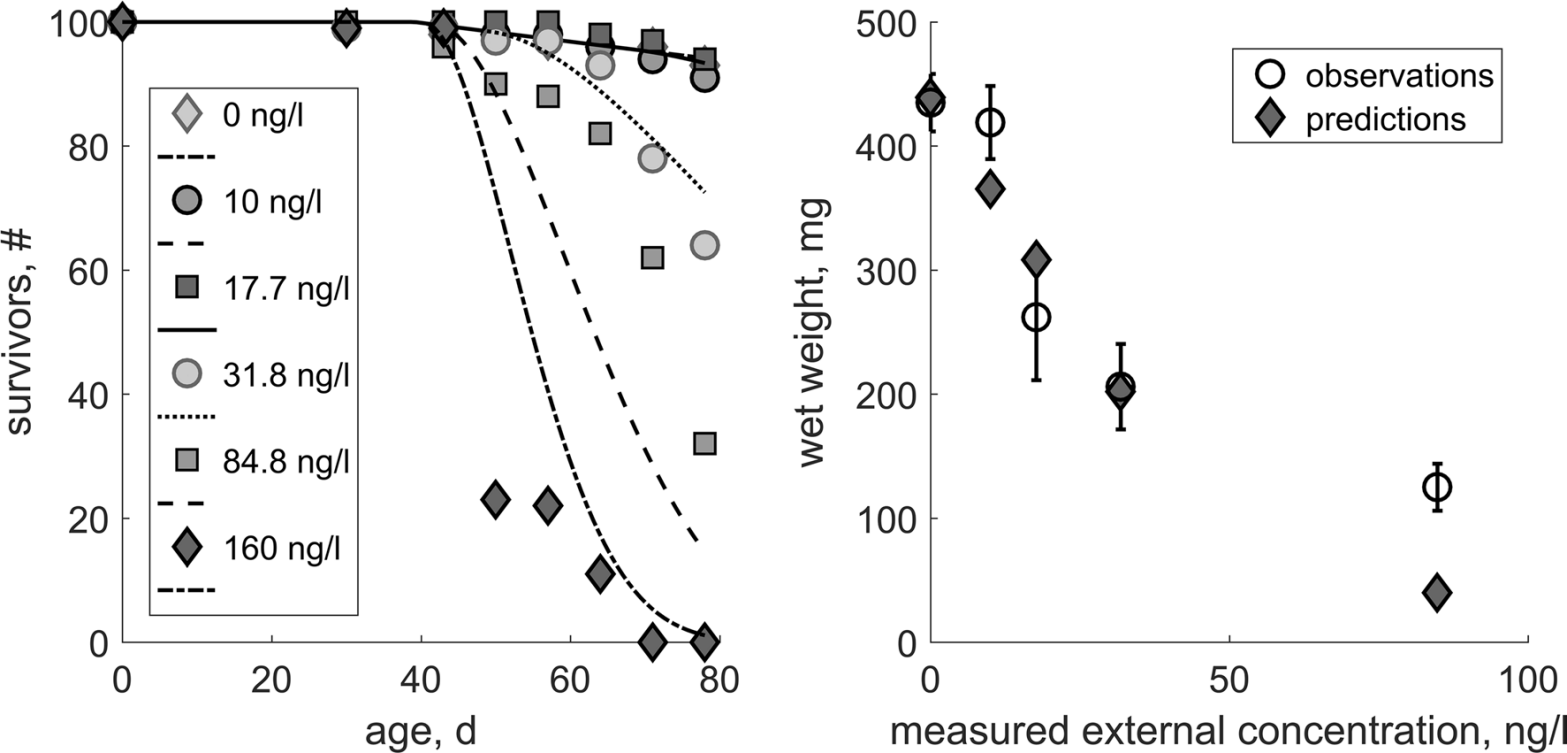
Calibration of the TKTD module. Comparison between model results and observations in Experiment 1 (the constant-exposure ELS study which was used to calibrate the TKTD module) for survival over time (right) and wet weight at the end of the test (left). Error bars represent the standard deviation

We fitted the TKTD parameters to the constant-exposure data (Experiment 1). Corresponding parameter values can be found in Table 1. A comparison between the predictions and empirical data is shown in Fig. 1. The resulting relative errors (RE; i.e. the mean of relative differences between model predictions and the data used for model calibration) compared with the empirical data are listed in Table 2. We use the RE to compare it to our previously defined performance criteria to evaluate the performance of the model (see Table 2).

**Table 1:** TKTD parameters and study-specific scaled functional response in Experiments 1 and 2

Symbol	Unit	DEB definition	Value
$\dot{h}_0$	d^−1^	Background hazard rate	0.001545
$\dot{k}_e$	d^−1^	Elimination rate constant	0.0001283
$c_0$	ng/L	Threshold concentration for effects on feeding	2.282e−09
$c_T$	ng/L	Tolerance concentration	2.159
$c_{0\text{s}}$	ng/L	Threshold concentration for effects on survival	0.6908
*b*	d^−1^	Killing rate	0.01965
$f_{\text{cW0}}$	−	Scaled functional response for Experiment 1	0.48
$f_{\text{A}}$	−	Scaled functional response for cohort A Experiment 2	0.73
$f_{\text{B}}$	−	Scaled functional response for cohort B Experiment 2	0.53
$f_{\text{C}}$	−	Scaled functional response for cohort C Experiment 2	0.73

The model outputs were generally very close to the empirical data (see Fig. 1). The difference for survival over time was with a 16.8% overestimation at 84.8 ng/L and 23.5% underestimation at 160 ng/L outside our performance criteria (Table 2). Simulated final wet weight deviated beyond the performance criteria for the concentrations of 17.7 ng/L (17.66% overestimation) and 160 ng/L (68.2% underestimation).

**Table 2:** Calibration: relative errors for the DEB model predictions when they are compared with the empirical data from Experiment 1 (the constant-exposure ELS study which was used to calibrate the TKTD module)

Treatment (ng/L)	RE for survival over time	RE for wet weight (test end)
0	0.006716	0.009802
10	0.009099	0.1277
17.7	0.01175	0.1766
31.8	0.02827	0.0191
84.8	0.1679	0.682
160	0.2347	n.a.^a^

^a^All individuals had died before the end of the test and have not been measured

### Validation of the DEB model (including the TKTD module): model predictions compared with data from Experiment 2

Results from Experiment 2 showed no beta-cyfluthrin-induced mortality in any of the cohorts. There were no sublethal effects for the cohort exposed as eggs (Cohort C). For the cohort exposed as alevins (Cohort B) the only sublethal effect was a 2-day delay in full onset of swim-up at 450 ng/L compared with the control (although completion of swim-up occurred at the same time as in the control). Swim-up fry (Cohort A) was clearly the most sensitive life stage based on the effects on feeding behaviour and clinical signs. The exposed fry showed an effect on feeding behaviour at 48 ng/L and above, which was reversible. There was no consequent effect on growth (weight and length) at 48 ng/L. Minor effects on growth (as length) were observed at 72 ng/L and above (see Fig. 4 and Additional file 1: Table S6) with a mean length 3.4% less than the control at 72 ng/L and a mean length 6.4% less than the control at 450 ng/L. The highest test concentration (450 ng/L) was the only one that induced clinical signs (e.g. loss of equilibrium). The overall no-observed effect concentration (NOEC) was a nominal peak concentration of 32 ng/L, based on the effect on feeding behaviour at 48 ng/L. The NOEC for growth (as length) was 48 ng/L.

**Fig. 2:**
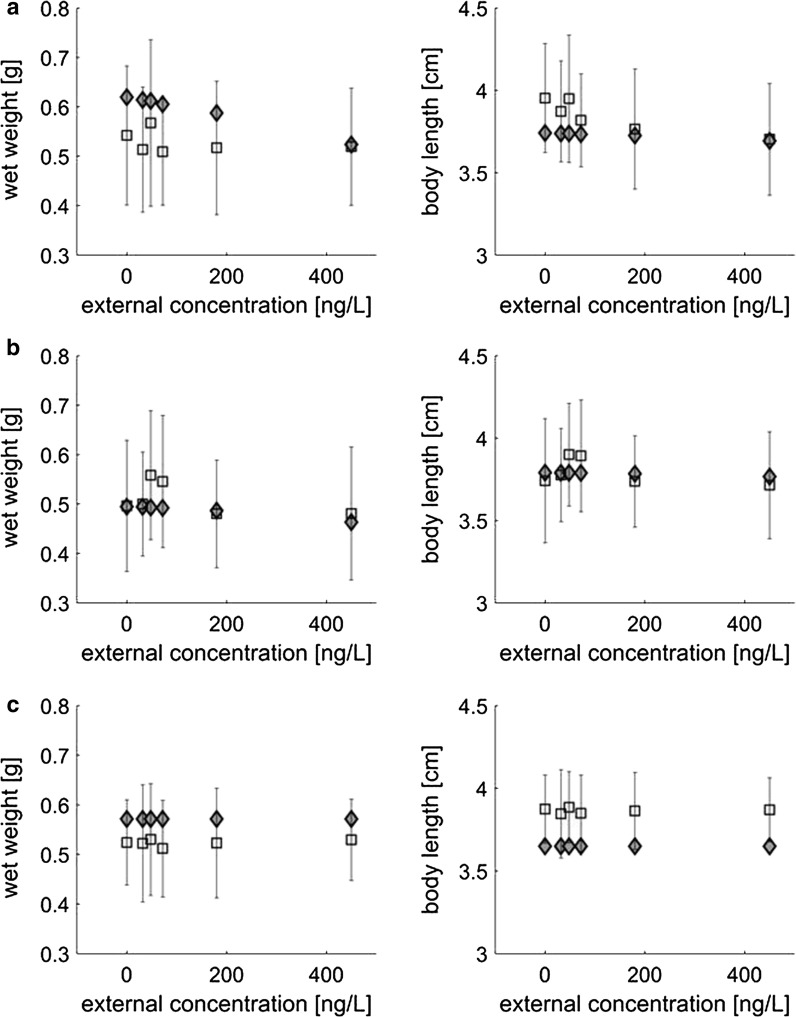
Results for Cohorts** a**–** c** for Experiment 2. Comparison between predicted and empirical weight (left) and length (right). Empirical data are only available for the end of assessment period (squared boxes with error bars, representing the standard deviation of the data). The grey diamond-shaped markers represent the model predictions

For the three experimental cohorts in Experiment 2, different food levels were estimated (see Table 1). The model predictions of weight, length and survival for Experiment 2 were compared to the empirical data. No significant effect on survival was recorded during the experiment, so we focus the comparison on weight and length at the end of the experiment (see Fig. 2). The simulations over time for all cohorts are also shown in Additional file 1: Figures S7–S9. The relative error for the comparison between experimental data and model predictions is shown in Table 3.

**Table 3:** Validation: relative errors for the DEB model predictions when they are compared with the empirical data from Experiment 2 (the peak exposure ELS study which was used to validate the TKTD module)

Treatment (ng/L)	Cohort A		Cohort B		Cohort C	
	RE for length (test end)	RE for wet weight (test end)	RE for length (test end)	RE for wet weight (test end)	RE for length (test end)	RE for wet weight (test end)
0	0.1426	0.0537	0.0036	0.0129	0.0901	0.0581
32	0.1958	0.0346	0.0118	0.0035	0.0940	0.0510
48	0.0783	0.0537	0.1173	0.0287	0.0779	0.0608
72	0.1890	0.0221	0.0981	0.0268	0.1163	0.0520
180	0.1358	0.0107	0.0131	0.0124	0.0928	0.0554
450	0.0083	0.0027	0.0366	0.0139	0.0794	0.0571

The empirical data show a slightly shorter final length of exposed swim-up fry (Cohort A) compared to the control for exposure concentrations of 72 ng/L and above. In the model predictions, this slight reduction in growth is not captured in terms of length, but in terms of weight. No differences from the control were predicted for weight and length at all treatment levels for the fish exposed as alevins.

**Fig. 3:**
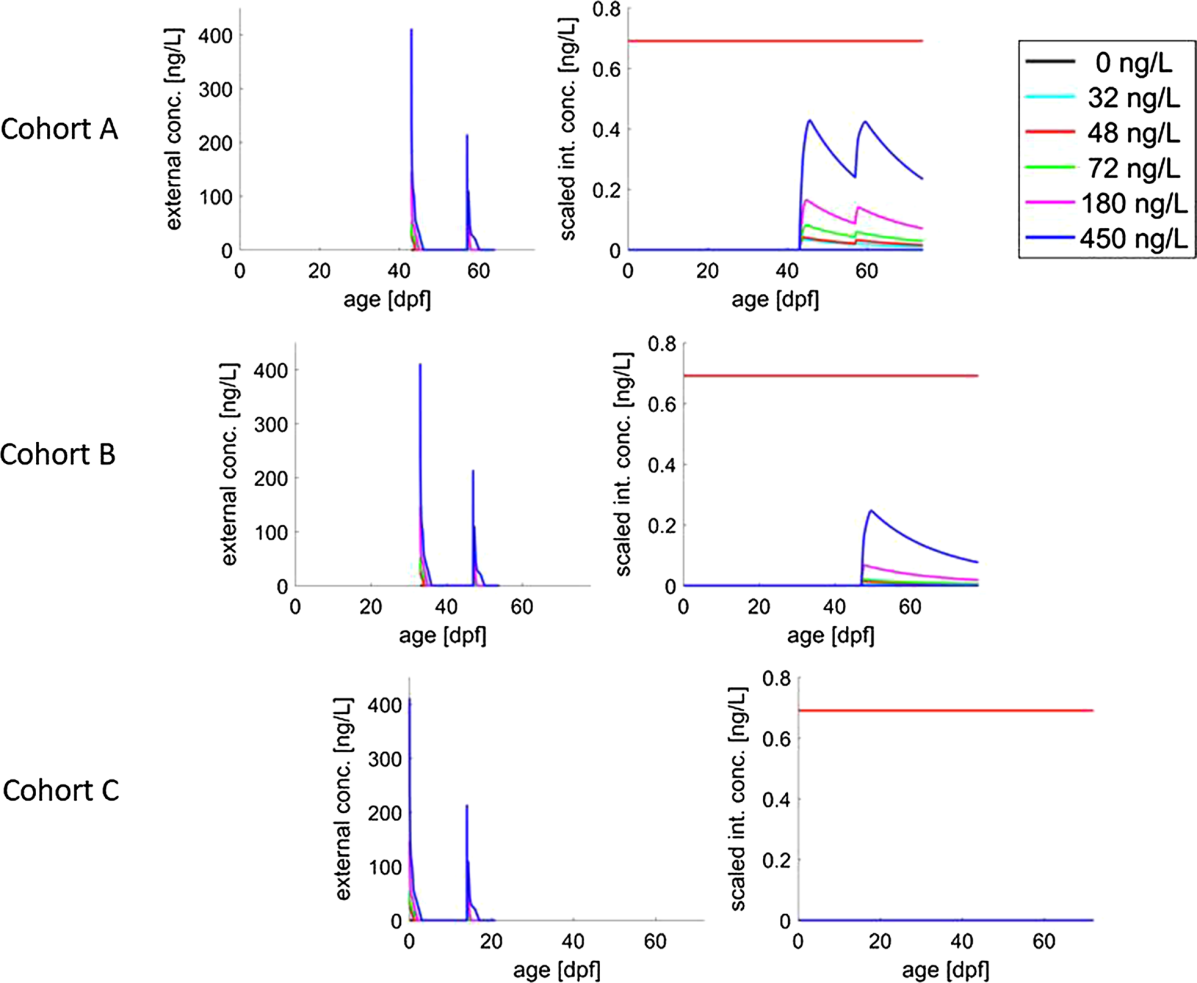
Predicted scaled internal concentrations in Experiment 2. The predicted scaled internal concentration of the three cohorts as predicted by the model. Following the assumption that the fish are only taking up the compound after swim-up (= the start of feeding), the three cohorts exhibit different internal concentrations. Cohort A takes up the compound directly, Cohort B only takes up the beta-cyfluthrin after the first pulse, and Cohort C does not take up the beta-cyfluthrin. The horizontal red line represents the no-effect threshold for lethal effects, which is not being passed by any of the cohorts, so that no effects on survival are predicted

Figure 3 illustrates the predicted internal concentration for all cohorts. The threshold for ‘effect on feeding’, $c_{0\text{S}}$, estimated from Experiment 1, is only slightly above 0 ng/L. For all the cohorts, the predicted internal concentrations did not exceed the threshold (red line) for lethal effects, so the model predicts no effects on survival up to the highest concentration.

## Discussion

### Model calibration and validation

The DEB model (with TKTD module) presented here was calibrated using a study where rainbow trout early life stages were constantly exposed to cyfluthrin (Experiment 1), and then used to predict effects where the early life stages were exposed to pulses of beta-cyfluthrin (the realistic worst case exposure profile based on the PECs, realized in Experiment 2). For some of the concentrations, the effects are overestimated, while for others the effects are underestimated during the calibration. We attribute this discrepancy to the biological variability of the test system. Even though model calibration to the data did not match our performance criteria for high test concentrations, performance was good during validation testing: the model predicted no effects for the experimental conditions tested in Experiment 2, which was in agreement with the observations. This is because the lower peak concentrations that were tested in the pulsed-exposure experiment (32, 48, 72 ng beta-cyfluthrin/L) were in same order of magnitude as the NOEC of the constant-exposure study (10 ng cyfluthrin/L). At such levels, the model performed well during calibration. The tested scenario was the worst case scenario, which is why higher peaks (for which the model could not be validated) are not expected to occur. Therefore, we conclude that the model can adequately predict independent data on the outcome for exposed eggs, alevins and swim-up fry, from both the qualitative (response pattern) and quantitative points of view under realistic exposure scenarios for beta-cyfluthrin.

During model validation, no differences from the control were predicted for weight and length at all treatment levels for the fish exposed as eggs (Cohort C), and very low level effects were predicted for the fish exposed as alevins (Cohort B). This is in full agreement with conclusions from empirical data. However, data availability for beta-cyfluthrin was not ideal for an in-depth model validation. The data set did not provide any information on effects (since there were hardly any observed) and can thus only be used to illustrate that the model is in agreement with the prediction of no effects. Having a validation study that shows effect would be preferable for further validation of the modelling approach. Moreover, TKTD model calibration and validation is best done using time-dependent data (e.g. [18]). In both ELS studies, the only information on effects on growth is body size measurements at the end of the test (as prescribed in the OECD guideline). For a DEB analysis, ELS studies would yield much more information if measurements on growth over time would be available. Even one additional data point (e.g. weight/length after hatching or at swim-up) would substantially increase the informative value of the data for a DEB analyses and thus the confidence in the subsequent model predictions. Following the recently published EFSA opinion on TKTD models (REF), validation data should comprise of at least two exposure profiles with at least 2 pulses each separated by a no-exposure interval of different duration. Moreover, mortality or immobility reported for at least 7 time points. If these rules are followed when conducting modified exposure studies, the data will be useful for the validation of TKTD models.

So far, unfortunately it was not possible to calculate 95% confidence intervals for the parameters using the modelling framework we chose here (Add-My-Pet-framework, AmP; see "Methods" section). Recently, a method to calculate these intervals has been developed and is in the testing phase [19]. Combining our modelling framework with the prediction of 95% confidence intervals when testing is completed will make this approach even more suitable for application in environmental risk assessment of chemicals.

### Mechanism of effect before swim-up

The results of the experiments and the modelling suggest that there is no significant effect of (beta-)cyfluthrin on eggs or alevins up to concentrations of 400 ng/L at constant exposure and 450 ng/L in a two-peak-exposure scenario. This may seem somewhat surprising, since most type I and type II pyrethroids have been found to cause mortality and developmental deformities in zebrafish embryos [20, 21]. It has even been suggested that by default, a potent insecticide exhibits high toxicity to fish [20]. On the other hand, high concentrations of fenvalerate (another type II pyrethroid) have previously shown to lead to delayed hatching in zebrafish eggs, but the larvae that hatched were not found to differ from the control [22]. This supports the finding of our study, which suggests that the eggs and alevins are not affected by the compound, and that they only experience the effects of the exposure after swim-up.

One hypothesis that could explain the observed pattern lies in the developmental process of the gills in the early life stages of the rainbow trout. The developing trout transition from full cutaneous respiration in the egg to full gill respiration as swim-up larvae (see, e.g. [23]). After hatching, respiration first takes place via the surface of the yolk sac, while the gills continue to develop. At feeding, gill development is completed, and respiration takes place fully through the gills. We hypothesize that only at the start of feeding, the uptake rate of the (beta-)cyfluthrin is fast enough to lead to effects. As a simplification, we thus assume that the uptake of the compound only starts when the fish have started to feed (as can be seen in Fig. 3).

### Mechanism of effect after swim-up

Effects of feeding impairment on growth have previously been deduced using a TKTD modelling approach for effects of imidacloprid in *Daphnia magna* [24]. In that study, the authors showed with the model that feeding impairment alone was responsible for observed effects on both growth and reproduction. Recently, it was suggested that the AOP framework has the potential to be used in combination with the DEB modelling framework to gain a better understanding of the mechanism of the underlying effects [25].

Pyrethroid insecticides have since long been known to be neurotoxic to fish and may lead to decreased swimming performance [20]. In a review on the development and application of the AOP framework for understanding and predicting chronic toxicity, Groh et al. [17] suggested that for pyrethroids locomotory abnormalities of fish may cause feeding impairment that may lead to reduction in growth. Following these findings, observations of feeding behaviour were included in Experiment 2. Indeed, during calibration of the TKTD module, it was found that the effects in Experiment 1 could be explained by assuming that exposure started at the time fry began to feed which happened approximately at day 43 (post-fertilization) under these experimental conditions. In Experiment 2, the organisms exposed as eggs (Cohort C) or as alevins (Cohort B) showed no impairment of feeding behaviour after the resulting fry completed swim-up and hence no effect on growth either (final length and weight). Thus, even though feeding behaviour was not monitored in Experiment 1, we conclude (i) that the observed effects on growth in Experiment 1 were a consequence of reduced feeding, and (ii) that this reduced feeding in Experiment 1 was probably a consequence of exposure of post-swim-up fry and not due to previous exposure of these organisms at a non-feeding life stage (i.e. as eggs or alevins).

### Lack of mortality and sublethal effects explained by the predicted (scaled) internal concentration

In the model, effects on biological endpoints were linked to the scaled internal concentration, which is presented in Fig. 3. For the simulation of Experiment 2, the predicted scaled internal concentration of beta-cyfluthrin remained below the no-effect concentration for survival (red line) for all cohorts, which is why no compound-induced mortality was predicted by the model. For modelled ‘effects on feeding’, the highest scaled internal peak concentrations were greater than the threshold for effects (blue line; almost zero) in Cohort A and Cohort B, which led to predictions of a slight effect on growth. In reality, in Experiment 2, there were no effects on feeding behaviour or growth at all concentrations for Cohort B and at 32 ng/L for Cohort A. We hypothesize that due to the short duration of the (experienced) exposure and fast internal metabolism, the compound did not induce effects on feeding behaviour and growth in these cases.

### Effects on growth: length vs weight

In the validation, the model predicted a reduction in weight (Cohort A). However, the actual experimental results showed a slight reduction in length and no significant effects on weight. In the model, predictions for weight are a combination of structure and reserve (see Additional file 1). Effects on feeding first have an effect on the reserve density, which in turn directly affects weight. Predictions for length are affected when the resources normally used for growth are redirected to other processes, which will happen when the available reserves are not sufficient to cover both maintenance and growth. The data on weight at the end of Experiment 2 show a higher variability than the data on length, which may be caused by biological variability. Effects on length were found to be significant in the highest concentrations, but the variability in the weight measurements may have been too large to allow for a determination of effects on weight. The model predicted slight effects on weight for the exposed swim-up fry (Cohort A) at 48 ng/L and above (Fig. 2). However, these predicted effects were not biologically relevant because they were within the range of the variability of the control measurements for weight. A similar effect pattern with even lower intensity was predicted for the alevins (Cohort B).

The model predicted a slight reduction in growth at the highest peak concentration (450 ng/L). Despite this growth reduction being in the range of the control variability, it could still be predicted by the model. This shows that the model was able to predict the onset of effects on growth, even though the predicted endpoint was not the same. The model predicted that a 16.43% lower growth than the control would occur for exposure peaks of 450 ng/L for swim-up fry. In reality, Experiment 2 showed a 6.4% lower growth than the control at this concentration. Therefore, the model prediction was slightly more conservative than the actual empirical results. Based upon this, we are confident about the predictive power of the model and its relevance to ecotoxicological risk assessment.

## Conclusions

We here used a DEB model to (i) test the hypothesis that the growth effects in Experiment 1 and Experiment 2 can be explained by impaired feeding, and (ii) mechanistically investigate and explain the differences between the observed effects in the two studies. We have shown here that the basic physiological model and the AmP parameter set is suitable to describe development of rainbow trout in the control of ELS (if the food level is adjusted to experimental conditions). The modelling exercise illustrates that a DEB model in combination with a TKTD module can enhance the mechanistic understanding of ecotoxicological effect studies by helping to identify the mechanistic mode of action. This approach has the potential to greatly increase the utility of existing ecotoxicological studies for environmental risk assessment because effects can be simulated for a large amount of time-variable exposure profiles (e.g. from FOCUS modelling). Requirements to do so are a constant exposure study for model calibration and a modified-exposure study for model validation. However, existing test protocols could be improved to increase the value of the resulting experiments for TKTD model calibration and validation. Experiment 2 would have been more informative for validation if higher concentrations were tested to increase visualization of effects. Moreover, multiple measurements of endpoints throughout the experiment (rather then only at the end) would allow capturing dynamics of effects and thus increase certainty of good model performance. With the DEB model (with TKTD module), we were able to show that the results from Experiment 1 and Experiment 2 were consistent with each other. The model was able to explain the basis of the difference in the observed results, in terms of prediction of internal concentrations for the two different exposure profiles. As such, the modelling can increase confidence in the use of higher tier modified-exposure studies. Due to the generic nature of the model, this approach has the potential to be used for any combination of test organism and test substance.

## Methods

### The DEB model and physiological parameters

Three decades of research into DEB theory has resulted in a database of physiological parameter sets for 1032 organisms ([26], as of 2018/02/18). These parameter sets describe the energetic fluxes within individuals from energy uptake and energy distribution to maintenance, growth, and reproduction. The parameter sets are freely available [26], and can be used in combination with a well-tested Matlab code that has frequently been used (see, e.g. the recent 6th special issue on DEB theory, [27]). A detailed description of the DEB model can be found in Additional file 1. Overall, model outputs are predictions for, e.g. the weight, length and number of offspring along the life cycle of an individual, as well as the survival probability within a group of individuals. Some of the model outputs are directly relevant to ecological risk assessment (e.g. survival and growth, which are directly connected to the protection goals for rainbow trout), while some others contribute to a better understanding of the physiological state and stress level of organisms (e.g. energy reserves, respiration, feeding status). If no detailed information on food intake is available, the so-called scaled functional response* f* (in between 0 and 1) is used to represent the food level. Hereby, 1 represents ad libitum feeding, and 0 no food.

In the DEB model, transitions from one life stage to another are captured by maturation; a certain level of maturity has to be reached for the organism to transition to the next stage. The transition from one life stage to the next is usually accompanied by slight changes in the energy allocation. For example, in the present study, we consider eggs, alevins and swim-ups. In the egg stage, organisms do not feed externally but get their energy from reserves in the egg. After reaching a certain maturity level (hatching), the fish transitions to the alevin stage, where the organisms are still not feeding, but get energy from the yolk sac. After reaching another maturity level (‘birth’ in the DEB model), the organism transitions to the swim-up stage (‘juvenile stage’ in the DEB model) and starts to feed externally. In this current modelling study, we investigated effects in two ELS tests on rainbow trout. Such tests do not include reproduction as an endpoint. Hence, model outputs related to reproduction are not applicable.

### The Add-My-Pet tool and -database

Rainbow trout parameters were taken from the Add-My-Pet (AmP) database ([26], Version 20170527). Note that an updated version of parameters has been published following the completion of our modelling study. Changes in parameters do not influence the conclusions drawn from our study. The main difference in the parameters is in the maturity levels for the switches in life stages. As our study concludes that all effects of (beta-)cyfluthrin derive from feeding impairment only and not from direct effects on maturation use of the new parameter set would not generate different results as all other parameters deviate only slightly in the new version. The version we used can be accessed from the AmP website via version control.

For all AmP entries, published data from the literature is used for parameterization. On the AmP website, the so-called AmP-tool is provided, which allows for the estimation of parameters with a well-tested code. Parameter estimation is done based on the minimization of a parameter-free loss function [28, 29]. This function takes the different dimensions of all data sets into account simultaneously keeping all other parameters constant, without the need for additional parameters. The minimum is found using a Nelder–Mead simplex method; a simplex is a set of parameter sets with a number of elements that is one more than the number of free parameters. The specified initial parameter set, the seed, is one of the elements in the simplex, and the others are generated automatically in its ‘neighbourhood’. The simplex method aims to replace the worst parameter set by one that is better than the best one within the set, i.e. gives a smaller value of the loss function. During the procedure, the parameters can be filtered to avoid the combinations of values outside their logical domain [30].

Data usage, model code and parameters are routinely evaluated and tested by the database curators before publication of an entry on the Add-My-Pet database. We conducted a further evaluation of the used code and parameters of the rainbow trout using specific model performance criteria.

### Assessing the performance of the physiological model

For the ELS studies under consideration of the present study, the most relevant endpoints are weight and length at the end of a test. Historical control data of ELS studies not used for model parameterization (Bayer database, unpublished) show that data on final body weight and length usually have a standard deviation of up to 10% for weight and up to 15% for length. Since the most relevant endpoints for our study are length and weight at test end, we decided to use the 15% deviation as model performance criteria for all predicted endpoints (Additional file 1: Table S2). The general model performance of the physiological model was evaluated by deriving the MRE. The data from 13 publications that were used for calibration, together with the RE, are listed in Additional file 1: Tables S2, S3. Corresponding model predictions are shown in Additional file 1: Figures S1, S2.

The data used for model parameterization stem from experiments from the literature studies that were conducted at different temperatures and feeding conditions (Additional file 1: Tables S2, S3). Based on this variability in conditions, and the fact that the DEB model performed well with these parameters, a good model performance can be expected from 2 to 18 °C and for various food conditions.

Details on model equations and an overview of the parameter values used can be found in Additional file 1.

### The TKTD module

In the DEB model, effects of toxicants can be included as a disturbance from the normal energy balance and allocation (i.e. under control conditions). These disturbances are included as changes in the DEB parameters that define these fluxes. Any compound can affect one or more of these parameters. What set of parameters is adapted to account for flux disturbances is referred to as ‘metabolic mode of action’. Potential metabolic modes of action are effects on assimilation, growth, maintenance costs, reproductive output and/or survival (e.g. [7, 8]). Using a linear differential equation, the TKTD module converts the concentration of the toxicant (the ‘external concentration’) to a resulting concentration of the toxicant within the organism (the ‘scaled internal concentration’). We use the scaled internal concentration (scaled by the bioconcentration factor) to reduce the number of parameters that need to be estimated. This concentration has the unit of external concentration, and the module only has one parameter to describe uptake and elimination of the compound [8]. The module provides a link of internal concentrations to effects. The link of external concentration to effects is equivalent to the more commonly known generalized unified threshold model for survival (GUTS, [18]), with the novelty of extending this method to sublethal effects. Compound effects can thus be included into a DEB model by adding a TKTD module that links internal concentrations to metabolic modes of action. This enables separation of species-specific and compound-specific parameters. Thereby the species model is generic and parameterization should not change for a specific compound. Thus, only the TKTD parameters are needed to be calibrated using experimental data because we have already shown that the DEB model for the rainbow trout fulfils our model performance criterion. The equations for the TKTD module have previously been published [8] and can be found in Additional file 1.

### Experimental data

**Fig. 4:**
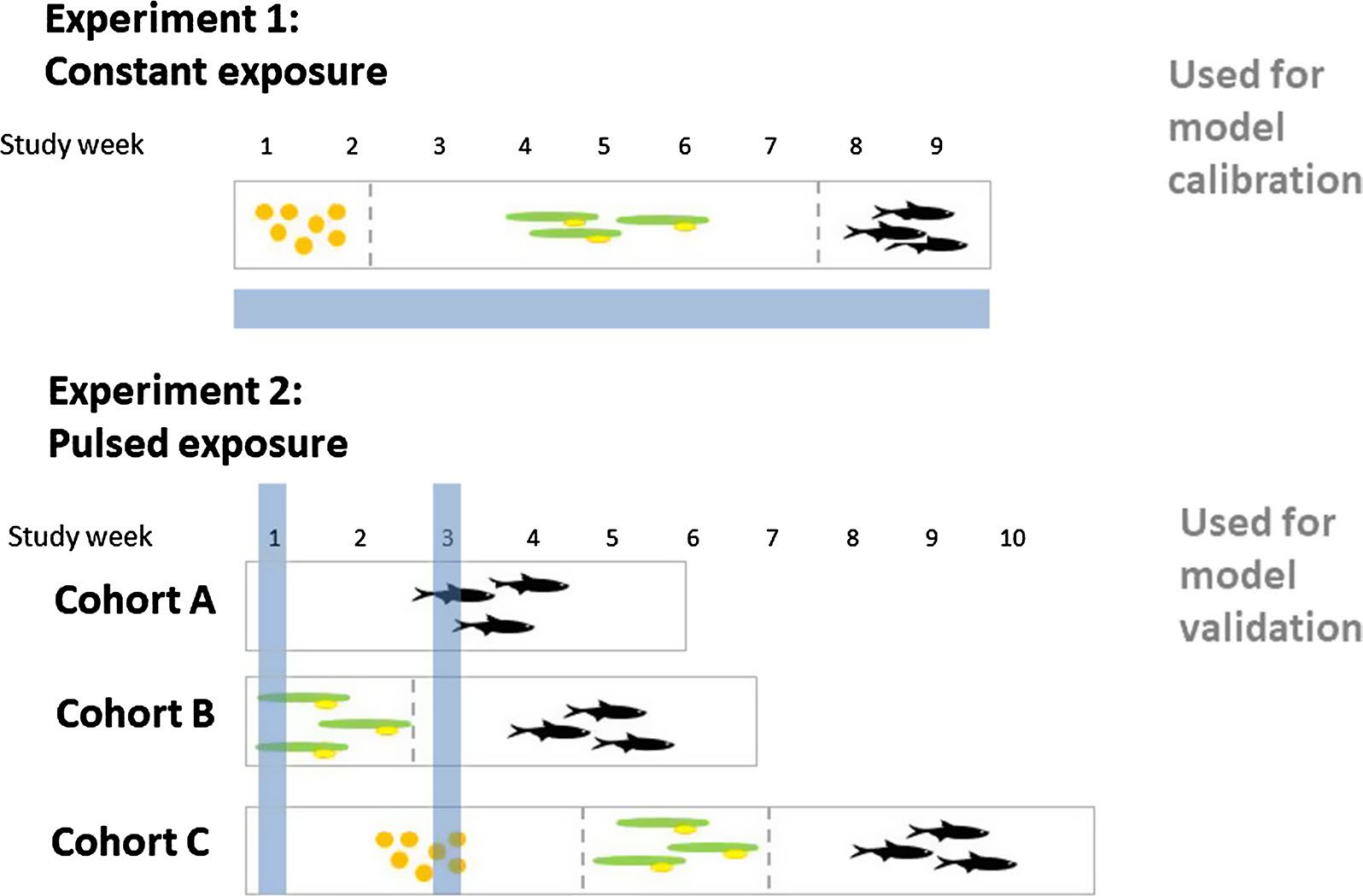
Comparison between Experiments 1 and 2. Dashed lines represent stage transitions from egg to alevin, and from alevin to swim-up stage. The blue bars represent the timing of exposure to the test substance (constant in Experiment 1, peaked in Experiment 2)

Experimental data used were derived from unpublished experiments that were used in the risk assessment of beta-cyfluthrin. A detailed description of both experiments can be found in Additional file 1; the main important details of the methods and the main results of the experiments are summarized below. The difference between the two ELS studies used is illustrated in Fig. 4.

#### Calibration data set: Experiment 1

As a calibration data set for the TKTD module, a constant-exposure ELS study was used [Experiment 1]. In Experiment 1, rainbow trout, starting as newly fertilized eggs, were exposed to mean measured (mm) test concentrations of 10, 17.7, 31.8, 84.8 and 160 ng cyfluthrin/L and monitored for 58 days at constant exposure. Survival was recorded weekly, and wet weight was measured at the end of the experiment. The weights at the end of the test as well as survival over time are reported in Additional file 1: Tables S4, S5). In total, 100 individuals were exposed (20 per concentration tested split into two replicates each). We chose to use the total number of survivors for modelling to remove effects of having two replicates. This experiment revealed that mortality at the highest concentration tested started at 30 days after the start of exposure (after swim-up of the fry), with a rapid increase within few days (see Additional file 1: Table S5). Organisms surviving the whole study duration had a concentration-dependent lower weight than the control organisms. Additionally, behavioural abnormalities were recorded but their nature was not specified in the report. The experimental data demonstrated no effects when the organisms were eggs and alevins (both of which are sedentary and do not feed). The NOEC for observed behaviour, final body weight, and survival derived from this study was 10 ng/L.

#### Validation data set: Experiment 2

In a peak-exposure experiment [Experiment 2], five different peak concentrations were studied in a realistic worst case two-pulse profile with peaks of 32, 48, 72, 180 and 450 ng beta-cyfluthrin/L (nominal concentrations). The exposure profiles were derived from the different PEC profiles. The two peaks occurred at a 14-days time interval. After each peak, the beta-cyfluthrin dissipated from the water column, with a DT50 of around 4 h.

Three different early life stages were exposed to beta-cyfluthrin and then followed until a minimum of 14 days after full establishment of free feeding of the swim-up fry stage. For an overview, see Additional file 1: Figure S3. To ensure that in the early life stages each received both pulses of exposure, the three life stages were exposed simultaneously within a single test system. Hence, the test began with groups of newly fertilized eggs (called ‘Cohort C’), alevins (called ‘Cohort B’), and early-post-swim-up fry (called ‘Cohort A’). There were four replicate test systems for the control and the five treatment levels.

Test endpoints were hatching rate, time to reach the swim-up stage, feeding behaviour after swim-up, clinical signs, survival, and weight and length at the end of the test.

### Calibration of the TKTD module using Experiment 1

We incorporated the TKTD module into the previously described AmP-code of the DEB model [26] for the parameter estimation of the TKTD module and followed a stepwise approach during calibration. Parameter estimates are based on simultaneous minimization of a weighted sum of squared deviations between the data set and model predictions (i.e. ‘loss function’) [30].

Before the parameterization of the TKTD module, the basic DEB parameters for rainbow trout and the control treatment data from Experiment 1 were used to estimate the food availability in that experiment by estimating the scaled functional response. Subsequently, we estimated the background hazard rate $\dot{h}_0$ using the mean survival of the control and of the concentrations that showed no significant effect on survival (0, 10 and 17.7 ng/L). As a next step, the remaining five parameters for the TKTD module (see Table 1) were estimated simultaneously. During the TKTD parameter estimation, the observed onset of effect patterns could only be matched by assuming that the uptake of the compound started after the fish had started to feed (swim-up), and not before. This is a simplified assumption that will be further discussed in "Discussion".

When including toxicant effects in a DEB model, five modes of actions are commonly tested: effects on assimilation/feeding, effects on maintenance, effects on growth and maturation, and effects on costs for making eggs/direct hazard to offspring [8]. Based on the conclusions of Groh et al. [17] and the observations of Experiment 2, we chose ‘decrease of feeding’ as the mode of action during calibration. The same scaled internal concentration was used for both lethal and sublethal effects. However, different thresholds were needed to capture the delayed onset of lethal effects. As mentioned previously, we assume that the ecotoxicological effect of beta-cyfluthrin and cyfluthrin is the same. Thus, we do not apply a correction factor when extrapolating the effects from one to the other.

### Validation of the TKTD module using Experiment 2

For model validation, predictions using environmental conditions of Experiment 2 were compared to data obtained in this experiment.

As a first step, the scaled functional response* f* was adapted to match the final bodyweight and length of the control of the different cohorts in Experiment 2. Note that* f* only applies for the last life stage, the swim-up fry. This step ensures that control growth is set adequately in the model. Then, the measured exposure concentrations over time in Experiment 2 were used as an input to the TKTD module. Next, the DEB model (with the TKTD module) was run and the predictions of body weight and body length were compared with the empirical data from Experiment 2. To avoid making further assumptions for the initial conditions of the different cohorts, the simulations for all cohorts were initiated using newly fertilized eggs, and exposure timing was adapted accordingly.

## Additional file

**Additional file 1.** The Additional file includes additional information on the experimental data used in the modelling study, as well as the model equations and fits of the basic physiological model.

## Abbreviations

TKTD: toxicokinetic–toxicodynamic
GUTS: generalized unified threshold model for survival
DEB: dynamic energy budget
EFSA: European Food Safety Authority
ELS: early life stage
MRE: mean relative error
RE: relative error
AmP: Add-My-Pet
NOEC: no observed effect concentration
FOCUS: FOrum for the Co-ordination of pesticide fate models and their USe
OECD: Organisation for Economic Co-operation and Development
AOP: adverse-outcome pathway
DT50: time required for the disappearance of 50% of the applied substance
PEC: peak-exposure concentrations
