# Supplementary material for: Modelling effects of time-variable exposure to the pyrethroid beta-cyfluthrin on rainbow trout early life stages
Source: Environ Sci Eur. 2018 Sep 18;30(1):36. doi: 10.1186/s12302-018-0162-0 (PMC6153864; doi:10.1186/s12302-018-0162-0)
Supplement: Supplementary file 1 — Additional file 1. The Additional file includes additional information on the experimental data used in the modelling study, as well as the model equations and fits of the basic physiological model. [file 12302_2018_162_MOESM1_ESM.pdf]

Supplementary information to: Modelling effects of time-variable exposure to the pyrethroid beta-cyfluthrin on rainbow trout early life stages

Elke I. Zimmer[1]*, Thomas G. Preuss[2], Steve Norman[3], Barbara Minten[4] and Virginie Ducrot[2]

[1]* *ibacon GmbH, Arheilger Weg 17, 64380 Rodorf, Germany*

[2] *Bayer AG CropScience Division 40789 Monheim Am Rhein Germany*

[3] *RidgewayEco, Harwell Innovation Centre, Oxfordshire, OX11 0QG, UK*

[4] *ADAMA Deutschland GmbH, Edmund-Rumpler-Str. 6, 51149 Koeln*

* *Corresponding author: elke.zimmer@ibacon.com*

1. Supplementary information

1.1. The physiological model

1.1.1. Background

We use an available physiological model based on Dynamic Energy Budget (DEB) theory. The DEB theory has been developed by Bas Kooijman in 1979. Since then, the theory has been continuously developed and tested in a multitude of applications, ranging from aquaculture to ecotoxicology, from bacteria to wales, from individual to population, resulting in over 500 peer-reviewed publications (see [18]; regularly updated). Together with an international group of DEB researchers, Bas Kooijman has biannually organized DEB-online-courses since the year 2000. In 2009, for the first time a practical face-to-face course was held in addition to the online course, which was followed by the first international DEB symposium. In the same year, the Add-My-Pet-project [1] was started, aiming at providing a tool that facilitates the data collection and parameterization for various pet species. The AmP-project has delivered an online platform for data/parameter collection maintained by a board of curators. The AmP platform also provides matlab code for DEB parameter estimation for all species. The code is freely available (open source software) and collaboratively developed and maintained in AmP Github repositories. The AmP platform also provides toolboxes that facilitate DEB modelling i.e. applications in general (DEBtool) and tools specifically designed for the parameter estimation of the DEB model to organisms (AmP-tool). AmPtool and DEBtool are licensed under the Free Public License 1.0.0 (0BSD) which is an Open Source Initiative compliant license, are accessible to all via GitHub and have online manuals. Updates, dedicated workshops, bugs and related articles are reported to the user community via the deb@listes.univbrest.fr mailing list (currently 87 subscribers), as

well as the AmP Researchgate, twitter and Facebook pages. Finally, a mediawiki powered website (http://www.debtheory.org/wiki/) is maintained which specifies how the AmP database is organized and how to edit templates for a species and submit it to the collection. Applications of DEB theory, many of which use DEBtool, are regularly presented at the international DEB international symposiums since 2009. The course and symposium participants represent an ever widening international multi-disciplinary user group. In 2017, the online course had 122 participants, and 47 participants took part in the intensive practical course. The 2017 Symposium had 75 participants from 20 countries.

1.2. Model assumptions

The DEB theory describes the energy metabolism of organisms throughout their life cycle based on the law of conservatism of mass and energy. It provides a set of rules that determine how much energy organisms assimilate from food, and how this energy is allocated to growth, development, reproduction and maintenance. Energy from food is first assimilated into reserve. The main reason for having a reserve compartment is to include metabolic memory: since organisms naturally never experience constant food conditions, they need to be able to survive periods of food shortage without harm; they thus react slowly to changes in their feeding conditions. In the DEB theory, the reserve does not correspond to a particular type of tissue or molecule (e.g. lipids) in the animal (i.e. what is usually named reserves in animal physiology). Rather, the reserve is a virtual pool of energy and macromolecules: any compound that is used to fuel the metabolic need of the organism belongs to the reserve compartment. Using a unique virtual reserve compartment allows to keep quantitative track of the energy/macromolecules that are mobilized at the same time in order to fuel the different physiological functions. The DEB model has clear rules that establish priorities/trade-off in energy allocation to these functions. From the reserve, the mobilized energy is split into two fractions. A fraction κ goes into the somatic or growth flux, and a fraction (1-κ) goes into the maturation or reproduction flux: this κ-rule establishes the trade-off between the various life-history traits e.g. growth and reproduction. On both sides, first the costs for maintaining the current status (i.e. biomass and degree of maturation) are paid, and only after that, the remaining energy is used to build up new structure (i.e. biomass) or maturity (e.g. sexual development, enzymatic/immunologic toolkit). This rules establish that whatever its age, the animal gives priority to its survival, and only then invests in other life-history performances (grow, mature, reproduce). Generally, three life stages are considered in the DEB framework: embryo, juvenile and adult. The rules for energy acquisition and allocation are fixed along a development stage, but slightly vary between development stages. The transition to a new developmental stage coincides with a switch in energy allocation. For instance, the embryo is defined as an organism that does not feed and only lives from the reserves that were handed over from the mother. Once it starts feeding (first switch), it is considered a juvenile. When it reaches puberty and starts reproducing (second switch), it is considered an adult until it dies. Along the life cycle, metabolic activity produces damage to the body which forms the basis of the aging process in the DEB model; damage accumulation eventually leads to the death of the animal.

1.3. Notation

Before explaining the equations, we will shortly introduce the notation as it is suggested by Kooijman, 2010 [10]. Rates have dots, which indicate the dimension 'per time'. Analogous to the tradition in chemistry, quantities that are expressed per unit of structural volume have square brackets, [ ]. Quantities per unit of structural surface area have braces, { }.

1.4. Scales, state variables and parameters

The DEB model for rainbow trout operates at a daily time scale over the life cycle of the organism and its first generation of offspring. In the present study where we focus on individuals (or a small group of individuals), the spatial scale is that of a test vessel. These correspond to relevant time and space scales for ecological risk assessment at tier 1 and tier 2.

The model has three state variables: structural length L (cm), reserve E (J) and maturity E_H (J). Body mass is composed of structure (i.e. body tissues) and reserve (i.e. pool of energy and macromolecules not stored in a particular tissue), which allows to link model predictions for growth to different nutritional conditions of organisms. Maturity reflects the increase in complexity of the animal along its life cycle with regard to various aspects like enzymology, immunology, sexual development, etc. The state variable maturity E_H (J) captures the increase in complexity along the development in the form of energy invested into maturation. It is the internal clock that keeps track of the developmental stage of the organism: indeed, the amount of energy invested into maturation determines the switch from one life stage to another. Thus, the same DEB model can be used to model the whole life cycle of an organism, whereby differences between life stages exist. During the embryonic development, organisms do not feed ($f = 0$) or reproduce. When reaching the maturity threshold for birth (E_H^b), organisms start feeding and are considered as juveniles. Species that have a larval development start changing their metabolic rates after birth until they reach the maturity threshold for metamorphosis (E_H^j). After reaching the maturity threshold for reproduction i.e. puberty (E_H^p), organisms start reproducing and are considered as adults. The flux that was used for maturation (E_H) is then allocated to reproduction (E_R). The basic dynamics of the state variables are specified by the following equations (detailed in Kooijman, 2010): The dynamics of the state variables are specified by

$$
\begin{aligned}
\text{Reserve:} \quad \frac{d}{dt}E &= \dot{p}_A - \dot{p}_C \quad \text{if} \quad E_H > E_H^b \\
\text{Structural length:} \quad \frac{d}{dt}L &= \frac{\dot{r}}{3}L \\
\text{Maturity:} \quad \frac{d}{dt}E_H &= (1-\kappa)\dot{p}_C - \dot{k}_J E_H \quad \text{if} \quad E_H < E_H^p \\
\text{Reproduction buffer:} \quad \frac{d}{dt}E_R &= (1-\kappa)\dot{p}_C - \dot{k}_J E_H^p \quad \text{if} \quad E_H \geq E_H^p
\end{aligned}
$$

The mobilization flux $\dot{p}_C$, the assimilation flux $\dot{p}_A$, and the specific volumetric growth

rate $\dot{r}$ are given by

$$\begin{aligned} \dot{p}_C &= E(\dot{v}/L - \dot{r}) \\ \dot{p}_A &= f\{\dot{p}_{Am}\}L^2, \\ \text{and} \quad \dot{r} &= \frac{E\dot{v}/L^4 - [\dot{p}_M]/\kappa}{E/L^3 + [E_G]/\kappa}, \end{aligned}$$

The parameters $\dot{v}$, $\{\dot{p}_{Am}\}$, $[\dot{p}_M]$, κ and $[E_G]$ are explained in Table S1.

The so-called metabolic acceleration is a particular extension of the DEB model which is commonly applied in species that undergo a metamorphosis during early development ([10]; [2]), including rainbow trout. The motivation to develop this model extension was based on the fact that rainbow trout changes their shape in the early juvenile period, ended by metamorphosis. A change in shape alters the surface-area to volume ratio, and has an influence on all parameters that have length in their dimension: energy conductance and surface-area specific assimilation efficiency. The parameters increase proportional to length during the acceleration period, but stay constant before and after. As a result, growth at constant food is exponential after birth and changes into von Bertalanffy growth after (metabolic) metamorphosis. Consequently, the hatching time is altered with the extension: when using the extension, the energy conductance is lower during the embryonic stage than the adult, which leads to a prolongation of the predicted hatching time. This extension applies for most of the fish species listed in the Add-my-pet library and will be used in the present study with rainbow trout.

The mobilization flux $\dot{p}_C$, the assimilation flux $\dot{p}_A$, and the specific growth rate $\dot{r}$ are then modified to

$$\begin{aligned} \dot{p}_C &= E(\dot{v}\mathcal{M}(L)/L - \dot{r}) \\ \dot{p}_A &= f\{\dot{p}_{Am}\}\mathcal{M}(L)L^2, \\ \text{and} \quad \dot{r} &= \frac{E\dot{v}\mathcal{M}(L)/L^4 - [\dot{p}_M]/\kappa}{E/L^3 + [E_G]/\kappa} \end{aligned}$$

The shape correction function $\mathcal{M}(L)$ is given by:

$$\begin{aligned} \mathcal{M}(L) &= \frac{L_b}{L_b} \quad \text{if} \quad E_H \leq E_H^b && \text{(embryo)} \\ \mathcal{M}(L) &= \frac{L}{L_b} \quad \text{if} \quad E_H^b \leq E_H \leq E_H^j && \text{(early juvenile)} \\ \mathcal{M}(L) &= \frac{L_j}{L_b} \quad \text{if} \quad E_H > E_H^j && \text{(late juvenile)} \end{aligned}$$

Usually, metamorphosis is reached before puberty ($E_H^j < E_H^p$).

The core DEB parameters are explained in Table S1; the most relevant for our application are explained in more detail below. Since we are interested in growth and feeding behavior, one of the most important model parameter in our analysis is the scaled functional response

f. The scaled functional response f is the actual ingestion rate of an animal divided by the maximum ingestion rate for its size. For an individual under *ad libitum* feeding conditions, f = 1, whereas for a starving individual, f = 0, so that for limiting conditions 0 < f < 1. Other model parameters are described in details in Kooijman (2010).
Another very important parameter is κ. As mentioned above, κ determines the fraction of energy allocated to somatic maintenance and growth. Usually, κ is constant throughout the life cycle of an organism. A large κ (closer to 1) means that only a small fraction of energy is used for maturation and reproduction. A smaller κ means that less energy is used for growth, and more for maturation and reproduction. E.g., some fish species with several reproductive periods tend to have smaller values for κ, while mammals tend to have larger values. Because they relate to general physiological features, the three state variables of the DEB models (as well as most model parameters) are not directly measurable quantities. This means that we cannot get data on these state variables directly from experiments and vice versa: the values of these states variable over time do not provide direct information on the life-cycle traits of the animal. Examples are given below how the biological endpoints we want to predict (i.e. measurable quantities generally observed in laboratory or field test, and used as support for the risk assessment) can be calculated from the state variables and model parameters. In the DEB model, how much structure is built is calculated from the available energy. This structure is called structural length L. Structural length cannot be measured directly; however, it can easily be converted into measurable, physical length using the shape coefficient δ_M:

$$L_w(t) = \frac{L(t)}{\delta_M}$$

The parameter δ_M accounts for the various shapes of organisms, and is different for the same organism when different length measures are used. In this case study, we used the fork length as length measure.

Predictions for wet weight over time can be calculated by adding reserves and structural volume:

$$W(t) = L(t)^3(1 + e(t)\omega)$$

where ω is the contribution of reserve to weight, and e is the scaled reserve density, which is directly linked to the experienced food availability:

$$\frac{d}{dt}e = (f - e)\frac{\dot{v}}{L}$$

The scaled functional response f can be expressed as function of the scaled food level x, which is defined as the real food level X divided by the half-saturation coefficient K:

$$f = \frac{x}{x+1} \quad \text{with} \quad x = \frac{X}{K}$$

The scaled functional response thus includes information on the real food level and the half saturation coefficient. However, if no information on the exact amounts of food eaten are available, this function is treated as a parameter, and any variation on feeding behavior and

digestion or assimilation efficiencies then needs to be captured in this parameter. Thus, this parameter often needs to be adjusted if an experiment is modeled in detail. The number of offspring as a function of time can calculated from the energy flux going into reproduction E_R:

$$\dot{R} = \kappa_R \frac{d}{dt} \frac{E_R}{E_0}$$

where E_0 is the energy that is invested into one offspring, and κ_R is the so-called reproduction efficiency, which accounts for any costs related to the production of the offspring (this energy is lost during the process). Any predictions related to life-stage transitions (e.g. time to hatch, age at puberty) are calculated by simulating the life history of the organism over time. The zoom factor z can be used to compare body sizes in between species.

Table S1: Overview: parameters of the DEB model for rainbow trout (AmP version 20170527) at reference temperature (20 deg. C), state variables, fluxes and model outputs

Symbol	Value	Unit	Description
**Primary parameters: estimated**			
z	5.111	-	Zoom factor
δ_M	0.1014	-	shape correction coefficient (fork length)
$\{\dot{p}_{Am}\}$	3381.6	$J/d/cm^2$	max. surface area spec. assim. rate
$\dot{v}$	0.0486	cm/d	energy conductance
κ	0.56	-	allocation fraction to soma
κ_R	0.95	-	reproduction efficiency
$[\dot{p}_M]$	370.5	$J/d/cm^3$	vol-spec somatic maint
$\dot{k}_J$	0.002	1/d	maturity maint rate coefficient
$[E_G]$	5237.7	J/cm^3	spec cost for structure
E_H^h	1.65	J	maturity at hatching
E_H^b	5.74	J	maturity at birth
E_H^j	770	J	maturity at metam
E_H^p	488 10^4	J	maturity at puberty
**Other parameters**			
$\dot{r}$		-	volumetric growth rate
ω		-	contribution of reserve to structure
K		-	half saturation coefficient
**State variables**			
L		cm	structural length
E		J	energy reserve
E_H		J	energy invested in maturation
E_R		J	energy invested into reproduction
e		-	scaled reserve density
X			food density (not available here)
**Fluxes**			
$\dot{p}_A$		J	assimilation flux
$\dot{p}_C$		J	mobilization flux
**Model output**			
L_b		cm	structural length at birth
L_j		cm	structural length at metamorphosis
L_w		cm	physical length
$\dot{R}$		#	reproduction rate
E_0		J	energy costs for one egg

TKTD-module equations

The change in the scaled internal concentration follows a simple first order kinetic, driven by the elimination rate constant $\dot{k}_e$ and the external concentration c ([8]).

In growing organisms, the influence of size and the dilution by growth have to be accounted for:

$$\frac{dc_V}{dt} = \dot{k}_e \frac{L_m}{L}(c - c_V) - c_V \frac{3}{L}\frac{dL}{dt}$$

The hazard rate $\dot{h}$ is calculated from the difference between the scaled internal concentration and the no-effect threshold for survival c_{0s}. Note that we are only using this equation for the fish starting from the swimup-phase, since the eggs and alevins appear to not uptake the compound (see discussion). The effect intensity is defined by the killing rate $\dot{b}$:

$$\dot{h} = \dot{b}\max(c_V - c_{0s}, 0)$$

The change in survival probability S, which determines death, is given by adding the hazard caused by the compound to the background hazard rate h_0, and by integrating it over time:

$$\frac{dS}{dt} = -(\dot{h} + \dot{h}_0)S$$

Following the same principle as for effects on survival, the stress factor s is calculated from the difference between the scaled internal concentration and the no-effect threshold for sublethal effects c_0 .The effect intensity is defined by the so-called tolerance concentration c_T:

$$s = \frac{1}{c_T}\max(c_V - c_0, 0)$$

The stress factor s then reduces the energy taken up via feeding in the present case study, which is represented by the scaled functional response f, with f_0 representing the case without toxicant:

$$f = f_0(1 - s)$$

Effects on feeding reduce the energy uptake, and thus lead to growth reduction in the early life stages, and may lead to a reduction in reproduction in the adult stage.

1.5. Calibration data for the physiological model

The parameter values used in this project have been extracted from the AmP-database, and it is version 20170527. A new version has been uploaded before publication of the study presented here; however, even though the resulting TKTD parameters may turn out slightly different, the general conclusions of our study here are are not impacted.

Table S2: This table gives an overview of the uni-variate data (= observations over time e.g. growth curve) used for model calibration. The percentage deviation between the model prediction and the data larger than 15% is highlighted in bold. A deviation of 15% has been defined as model performance criterion based on data variability observed in historic ELS length and weight data. Initial conditions, food level and temperature refer to each individual data set that has been used for calibration.

Physiological process	Independent variable [unit]	Dependent variable [unit]	initial . cond.	food level f	T [C]	relative error	Reference
Growth	Time [d] 154 d	Wet weight [g]	1.54 g	0.4597	8.5	0.03541	[17]
Growth	Length [cm]	Wet weight[g]	-	1	-	0. 1044	[4]; [3]; [12]; [15]
Growth	Days post hatch [d]	fork length juveniles [cm];	1 g	1	-	0.05037;	[5]
Growth	Days post hatch [d]	fork length adults [cm];	1 g	1	-	0.04551;	[5]
Growth	Days post hatch [d]	wet weight juveniles [g];	1 g	1	-	**0.1668**;	[5]
Growth	Days post hatch [d]	wet weight adults [g];	1 g	1	-	0.06102	[5]
Ontogenetic development	Age [dpf] - 90 d	Dry weight of embryo [mg] Dry weight of yolk [mg]	egg	1	10	0.1265 0.08829	[11]
Ontogenetic development	Temperature [C]	Age at hatch [d]	egg	1	-	0.07111	[14]
Metabolic activity	Fork length [cm]	Wet weight [g], Oxygen consumption [μ mol/g/h]	-	1	-	**0.3894**	[9]
Metabolic activity	Temperature [C]	Oxygen consumption [μ mol/h]	-	1	-	**0.2458**;	[16]

The model fits of the DEB model to the calibration data shown in Table 2 are shown in Figure 1 and 2.

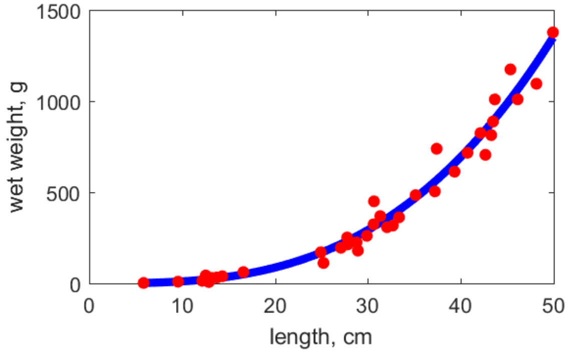

(a) Compiled growth curve [4, 3, 12, 15]

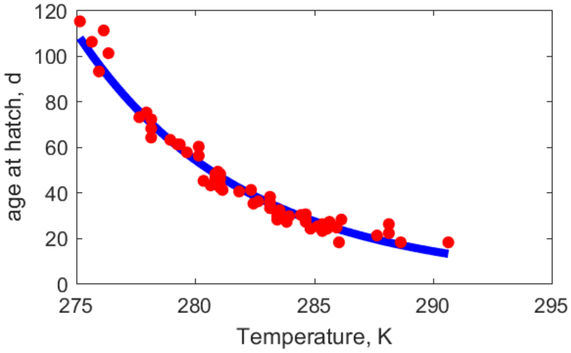

(b) Age at hatch at different temperatures [14]

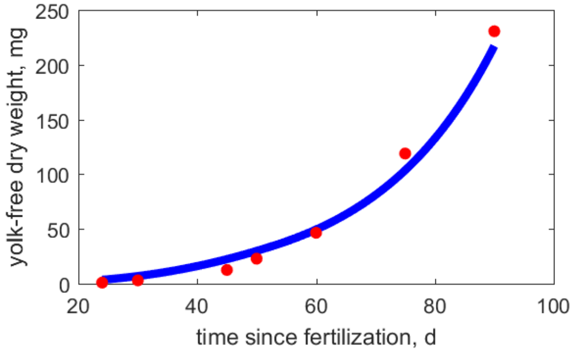

(c) Yolk-free dry weight during early development [11]

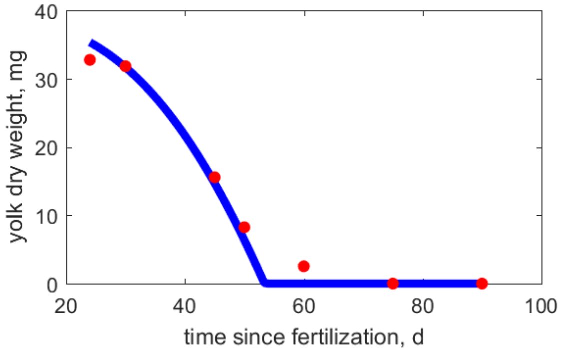

(d) Yolk dry weight during early development [11]

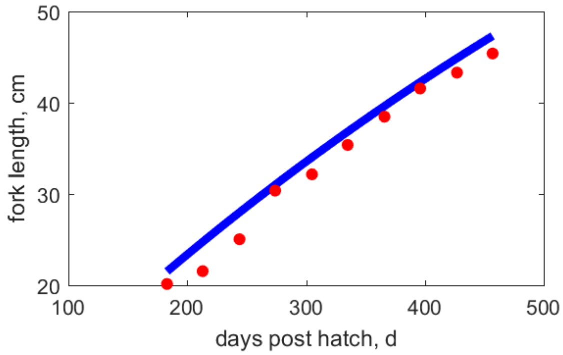

(e) Fork length as a function of age (first year trout, [5])

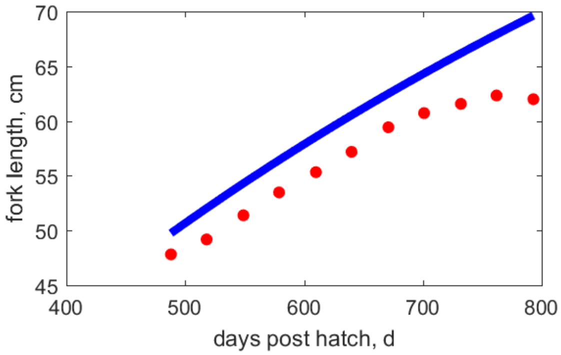

(f) Fork length as a function of age (second year trout, [5]

Figure S1: Calibration: the fits of the DEB model to various data (part 1). The blue lines denote the model prediction, and the red dots are the data points. A description of the data is presented in Table 2.

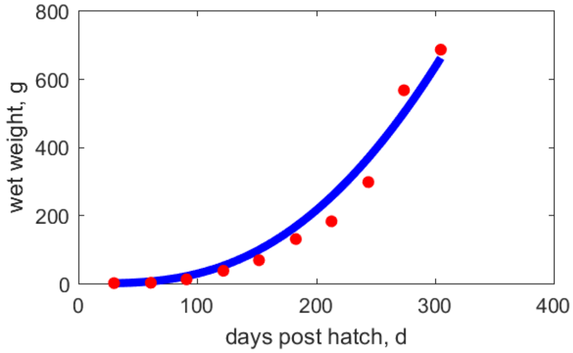

(a) Wet weight as a function of age [5]

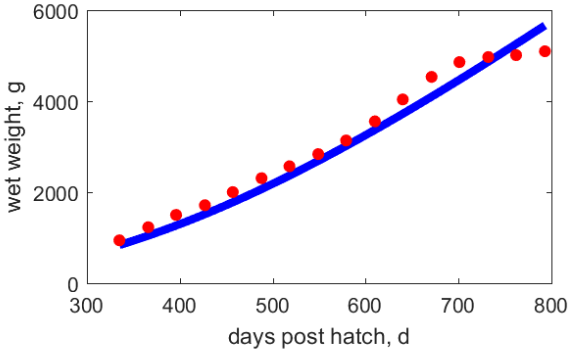

(b) Wet weight as a function of age (older trout, [5])

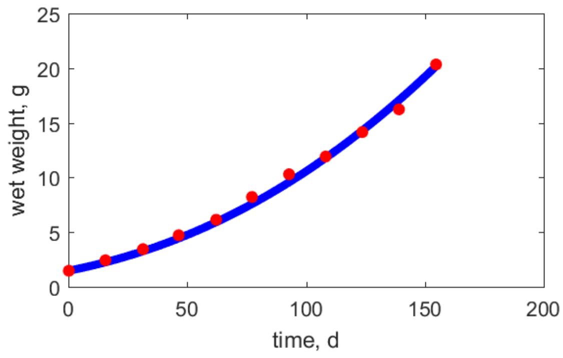

(c) Wet weight as function of age [17]

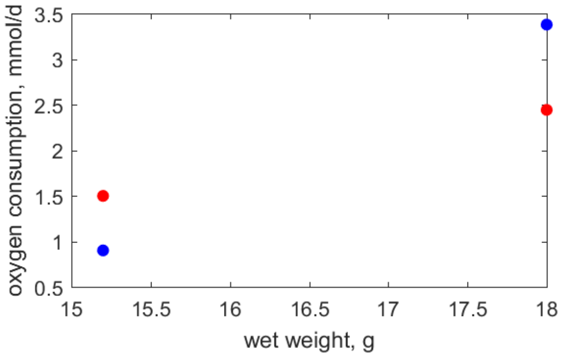

(d) Respiration as function of weight (larger trout, [9])

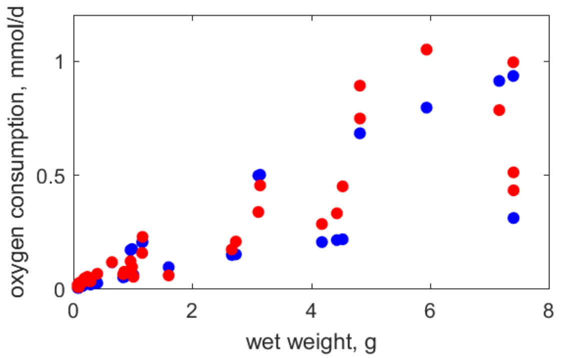

(e) Respiration as function of weight (smaller trout, [16])

Figure S2: Calibration: the fits of the DEB model to various data (part 2). The blue lines denote the model prediction, and the red dots are the data points. A description of the data is presented in Table 2.

Table S3: This table gives an overview over the zero-variate data (= single data points e.g. age at puberty) used in the calibration. All data in this table and table 2 were used simultaneously for model calibration. All food levels were set to f = 1. Note that information on temperature is only needed for data that relates to developmental times and rates.

Physiological process	Observation [unit]	Temp. [C]	Value	Prediction	relative error	Reference
Ontogenetic development	age at hatching [d]	10	32	35.09	0.09653	[7]
Ontogenetic development	age at hatching [d]	5	67	70.2	0.04782	[7]
Ontogenetic development	weight at hatch [g]	-	0.03879	0.03826	0.0137	[7]
Ontogenetic development	age at birth [d]	10	54	53.38	0.01142	[7]
Ontogenetic development	age at birth [d]	5	119	106.8	0.1025	[7]
Ontogenetic development	weight at birth [g]	-	0.03212	0.03572	0.1121	[7]
Ontogenetic development	age at puberty [d]	13	628	649.2	0.03377	[5]
Ontogenetic development	forked length at puberty [cm]	-	54	58.48	0.08295	[5]
Ontogenetic development	wet weight at puberty [g]	-	3500	3358	0.0407	[5]
Growth	ultimate total length [cm]	-	120	116.7	0.02751	[6]
Reproductive output	maximum reprod rate [$\#/d$]	12	174	174.8	0.004876	[13]
Reproductive output	dry egg weight [g]	-	0.0414	0.03921	0.05291	[7]
Metabolic activity	life span [d]	5	4015	4015	3.265e-06	[6]

1.6. Calibration data for the TKTD-module: Experiment 1

The calibration experiment was conducted from June-August 1985. Rainbow trout (Salmo gairdneri) eggs, incubated to the eyed stage, were supplied by Mount Lassen Trout Farms, Red Bluff, California. They were shipped on ice by air freight and then acclimated to the test temperature over a four-day period. During this period, eggs were examined for mortality. For each concentration, 100 eggs were divided between five test chambers in each of the two replicates. Two extra chambers per replicate contained approximately 50 eggs (fish) to be used as replacements for any test fish which escaped or were physically damaged during the study. The extra fish were reduced to 15 fish per replicate on day 25 of the study. Fish were fed Purina Trout Chow three times a day from swimup stage until the end of the study. Initially, size 00 feed was used and switched to size 1 as the fish grew larger. Each of 12 test vessels consisted of a 20-liter stainless steel tank with a perforated stainless steel tray divided into 12 incubation chambers. Individual replicates were identified by labels adjacent to each replicate. Labels contained chemical name, concentration and study number. Water was drawn from the bottom of each tank by a submersible pump and sprayed over the water surface through perforated stainless steel plates. A cooled, circulating water bath was used to control the temperature in the chambers. The bioassay water was municipal water, treated by passage through five activated carbon columns and cation resin bed. The chemical profile of this water was determined regularly. The temperature during the study ranged from 8.3 to 11.9 °C and the maximum variation between chambers at a given time was 2.5 °C. The water bath temperature was monitored continuously using a minimum/ maximum thermometer with attached probe. Both minimum and maximum temperatures were recorded once daily. The daily pH during the study period was 6.5 to 7.8. Once a week the test tanks were checked for buildup of foreign material. From the time feeding was initiated, the tanks were cleaned weekly. A 16 hour photo period was controlled by an automatic timer (Tork). All developmental stages (embryo, alevin and fry) were observed daily for hatch, swimup, mortality and signs of intoxication. Dead fish were removed and counted daily. Hatched fry and swimups were counted daily. On day 58, all survivors were sacrificed and weighed by chamber to the nearest milligram. The results are summarized in table S4 and S5.

Hatching occurred on average at day 10 of the experiment (see table S4). The experiment was started with eggs that were incubated to the eyed stage, but the exact age was not given. From From and Rasmussen, 1991 [7] we know that at 10 degrees, rainbow trout hatch at day 32 after fertilization. For the modelling, we thus assume that the eggs are 22 days old at the start of the experiment.

The mean concentrations, as determined by chemical analysis, ranged from 32 to 48% of the nominal concentrations and were used in toxicity calculation. The data indicate that under the test conditions only 32 to 48% of cyfluthrin (active ingredient) could be recovered.

Table S4: Overview of the data used for the calibration of the TKTD-module in Experiment 1.

Nominal conc (ng/l)	Mean actual conc.	Days to hatch	Total hatch	Total swimups	Survivors (test end)	Biomass per chamber (g)	Mean weight per fish (mg)
0	0	10	99	98	93	40.3	435
25	10	11	99	99	91	37.5	419
50	17.7	12	100	100	94	24.5	262
100	31.8	10	99	99	64	13.7	206
200	84.8	10	100	96	52	6.6	125
400	160	8	99	10	0	0	0

Table S5: The survival as a function of day of the experiment and nominal concentration. The numbers highlighted in bold show which data are significantly different from the control at the same day.

	Nominal concentration [ng/l]					
Day of exp.	0	25	50	100	200	400
10	99	99	100	99	100	99
23	98	99	100	99	96	99
30	98	98	100	97	90	**23**
37	97	98	100	97	88	**22**
44	96	96	98	93	**82**	**11**
51	96	94	97	**78**	**62**	**0**
58	93	91	94	**64**	**32**	**0**

1.7. Validation data: Experiment 2

The aim of this higher-tier laboratory study was to assess the effects of the active substance (a.s.) beta-cyfluthrin (tested as Bulldock 25 EC) on early life stages of rainbow trout (*Oncorhynchus mykiss*) with a realistic worst-case time-variable exposure-profile. The study was conducted in accordance with OECD Test Guideline No. 210 with adaptations in order to fulfil the objectives of the experiment. Rainbow trout was selected (from the species listed in the Test Guideline) due to its known sensitivity to beta-cyfluthrin. The study was intended to inform the higher-tier risk assessment for fish in the context of the EFSA Aquatic Guidance Document (GD; 2013). In accordance with Section 9.2.3 of this official guidance, this study is a refined exposure test with standard test species. A time-variable exposure profile was employed, representing two spray-drift events. This pulsed exposure consisted of two static dosing events in the presence of a ca. 10 mm layer of lake sediment. At other times there was a flow of clean water in order to maintain water quality. The dosing events were separated by 14 days. There were five test concentrations and a control group, all with four replicate test systems (tanks). The nominal peak exposure concentration for both dosing events for the five treatment levels were 0.032, 0.048, 0.072, 0.180 and 0.450 g a.s./L. A standard study on rainbow trout according to OECD TG 210 would include constant exposure of organisms developing from (i) embryos to (ii) alevins (i.e. sac-fry) to (iii) post-swim-up fry. In this higher-tier study, to ensure that these three early life stages each received both pulses of exposure, the three life stages were exposed simultaneously within a single test system. Hence, the test began with groups of newly-fertilised eggs, alevins, and early-post-swim-up fry. In the study these life-stage groups were referred to as eggs (cohort-C), alevins (cohort-B) and swim-ups (cohort-A), respectively. Within each test system, the life-stage groups were physically separated from each other within a single continuum of test medium (see Figure 3).

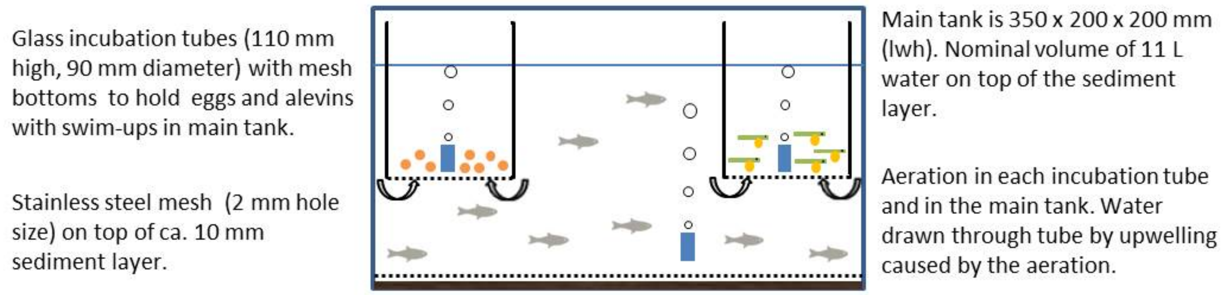

Figure S3: Diagram of exposure tank with three life stages in one tank kept separate but exposed to the same media. Lake sediment layer (ca. 10 mm thick) at the bottom of the tank with a stainless steel mesh barrier (ca. 25 mm above the sediment layer) to prevent fish from disturbing the sediment. The blue rectangles represent three airstones, which were in place to provide constant aeration and to ensure a homogeneous exposure medium.

For groups exposed as eggs (cohort-C), the study duration followed OECD TG 210. For groups exposed as alevins (cohort-B), to ensure a sufficient duration for expression of growth effects the study continued to 28 days after the 2nd dosing event. For groups exposed

as swim-ups (cohort-A), to ensure sufficient duration for expression of growth effects the study was continued to 14 days after the 2nd dosing event. These timings were selected to provide the most sensitive basis (biologically and statistically) for detecting effects on growth. Organisms were monitored for sub-lethal effects and mortality for the duration of the test.

Beta-cyfluthrin is a neurotoxicant with the potential to affect feeding behaviour. Hence, in addition to the standard measurement endpoints in OECD TG 210, feeding-behaviour of fish at the swim-up stage was also included. For the purposes of this study, a systematic method for assessing feeding-behaviour was established. Feeding-behaviour was assessed: in the exposed swim-ups (cohort-A) before and after each dosing event, and for cohort-B and cohort-C shortly after they had developed into swim-ups. Time-to-hatching and hatching success (cohort-C) and the time to swim-up (cohort-C and cohort-B) were recorded. Final body length and blotted wet weight of fish were determined for cohort-A, cohort-B and cohort C at the end of their respective phases. The biological data were analysed with CETISTM v 1.8.7.14 statistical software package. Water samples for chemical analysis were taken from test item and control test systems 0.33, 1, 4, 8, 16, 24, 48, 72 and 96 h after each dosing event and on the day before the 2nd dosing event. Concentrations of beta-cyfluthrin were determined using a GC-MS method of analysis with a Limit of Quantification (LOQ) of 0.01 g/L. The results of chemical analysis showed that the mean of the peak measured concentrations in the four replicates at each treatment level was within 20% of nominal at either dosing event 1 or 2. Therefore, the biological results were expressed in terms of nominal peak exposure concentrations. The analysed concentrations described the decline of beta-cyfluthrin in the water column over time. The mean of the measured concentrations at each time point for the four replicates tended to decline rapidly between 1 h and 4 h after dosing of the test system (see Figure 4).

The biological results showed that the early-post-swim-up stage (swim-ups, cohort-A) was clearly the most sensitive exposed life stage (see Table 6). This conclusion is based on the following observations for the exposed swim-ups (cohort-A): (i) a temporary impairment of feeding-behaviour (Active-feeding and Passive-feeding) at 0.048 g a.s./L and above; (ii) a slight but statistically significant shorter final body length than the controls at 0.072 g a.s./L and above (see Figure 5); and (iii) observation of clinical signs at 0.450 g a.s./L. Feeding-behaviour and final body length of swim-ups previously exposed as eggs (cohort-C) or as alevins (cohort-B) were unaffected at all test levels. Also, no significant treatment-related clinical signs were seen in cohort-C and cohort-B.

In conclusion, early-post-swim-up fry (referred to as swim-ups, cohort-A) was clearly the most sensitive exposed life stage in this study. The NOEC for two dosing events separated by 14 days was a nominal peak exposure concentration of 0.032 g a.s./L. This was based on an effect on feeding behaviour in exposed swim-ups (cohort-A) at 0.048 g a.s./L (the LOEC) and above. There was no consequent effect on growth (weight & length) at 0.048 g a.s./L. Hence, 0.048 g a.s./L is considered to be the overall No Observed Adverse Effect Concentration (NOAEC), at which there were no effects on time-to-hatch (cohort-C), hatching success (cohort-C), time-to-swim-up (cohort-C and cohort-B), incidence of clinical signs, growth and

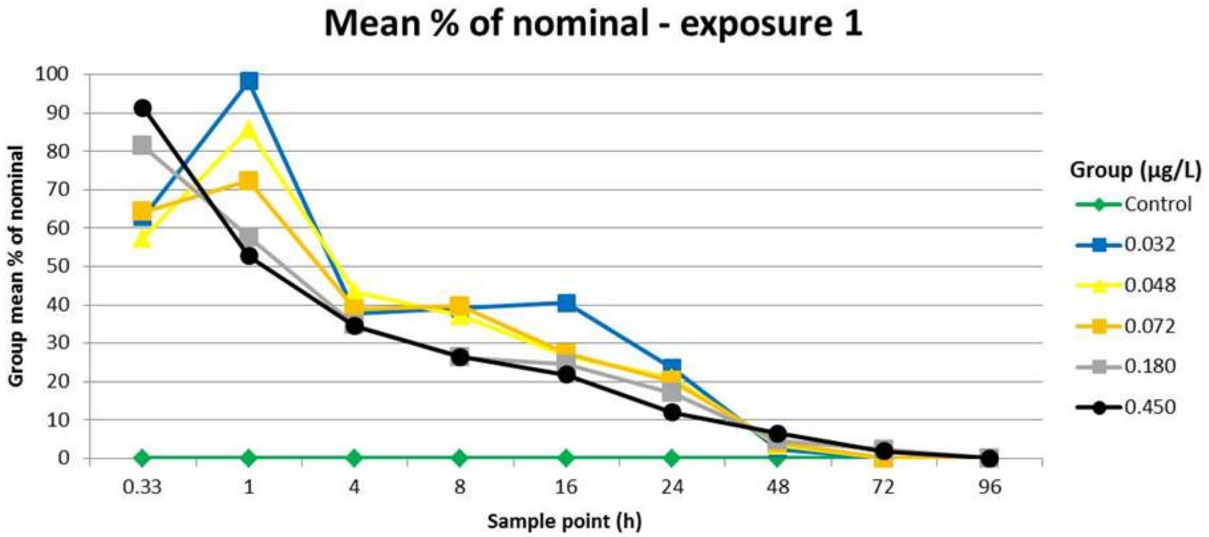

Figure S4: Analytical results for water for first exposure pulse in Experiment 2. These describe the exposure-profile. Results are stated in terms of the % of the intended (nominal) concentration. The DT50 was around 4 hours.

survival (cohort-C, cohort-B, and cohort-A) (these being the standard biological parameters assessed under OECD Test Guideline No. 210).

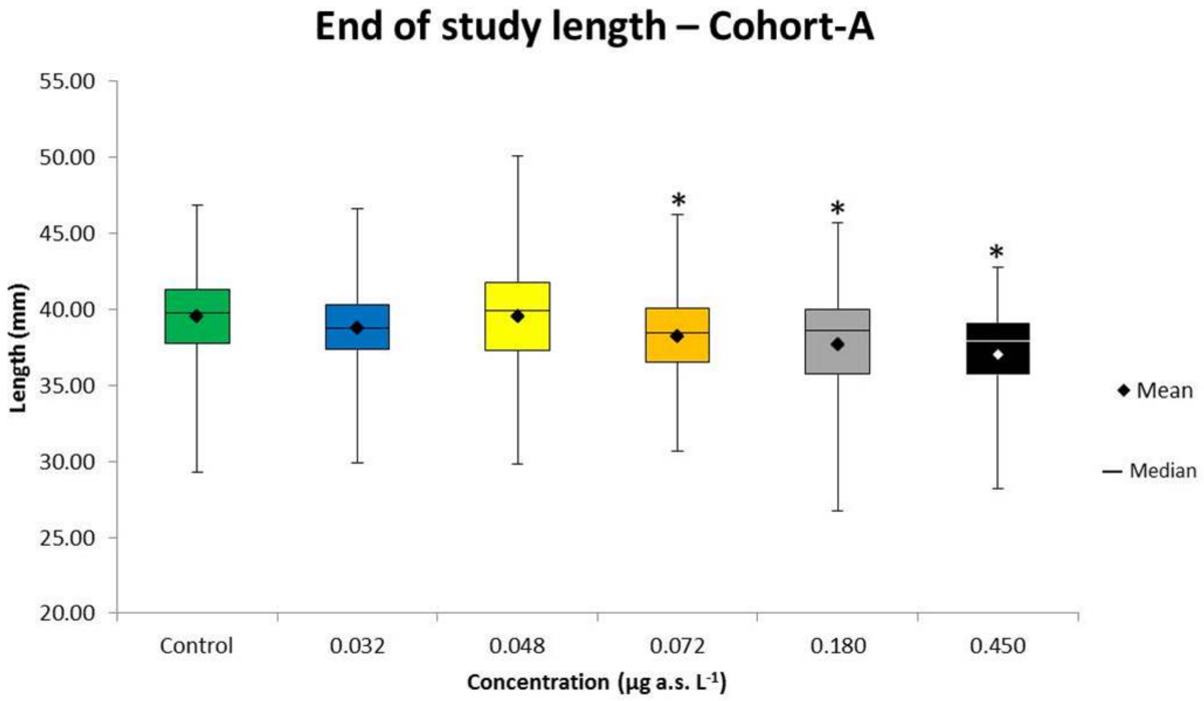

Figure S5: Fry length at end of study for cohort-A (Day 31). Box and whisker plots show mean, median, 2nd (upper box) and 3rd (lower box) quartiles and total range (maximum and minimum values, represented by the whiskers). * denotes statistically significant difference to control group ($P \leq 0.05$). The box above indicates whether a short-term effect on feeding behaviour was observed at the time of first (Day 0) and/or second (Day 14) exposure pulse.

Table S6: The wet weight and length measurements at the end of the test in Experiment 2, used for validation of the TKTD-module. The numbers in bold highlight which data are significantly different from the control at the same day (which is only cohort A, the three highest concentrations for length).

Treatment [ng/L]	A			B			C		
	wet weight [mg]	SD	% SD	wet weight [mg]	SD	% SD	wet weight [mg]	SD	% SD
control	542.0	140.7	25.96	496.2	132.7	26.74	524.1	85.8	16.37
32	513.4	126.6	24.66	499.9	105.2	21.04	522.2	118.1	22.62
48	567.4	168.5	29.70	558.3	130.4	23.36	530.0	112.6	21.25
72	508.9	107.9	21.20	545.5	133.7	24.51	511.8	97.6	19.07
180	517.0	135.0	26.11	480.2	108.8	22.66	522.8	110.4	21.12
450	519.3	118.7	22.86	480.8	134.7	28.02	529.3	81.8	15.45
	length [mm]	SD	% SD	length [mm]	SD	% SD	length [mm]	SD	% SD
control	39.53	3.31	8.37	37.41	3.77	10.08	38.74	2.06	5.32
32	38.72	3.06	7.90	37.76	2.83	7.49	38.45	2.67	6.94
48	39.49	3.87	9.80	39.00	3.12	8.00	38.85	2.15	5.53
72	**38.18**	2.82	7.39	38.92	3.39	8.71	38.49	2.31	6.00
180	**37.65**	3.65	9.69	37.37	2.77	7.41	38.63	2.33	6.03
450	**37.03**	3.40	9.18	37.14	3.24	8.72	38.70	1.93	4.99

1.8. Model validation

The model validation was conducted by comparing the model predictions with the model parameterized to Experiment 1, and only adjusting the conditions and duration to Experiment 2. The parameters for food level were adjusted to fit control growth.

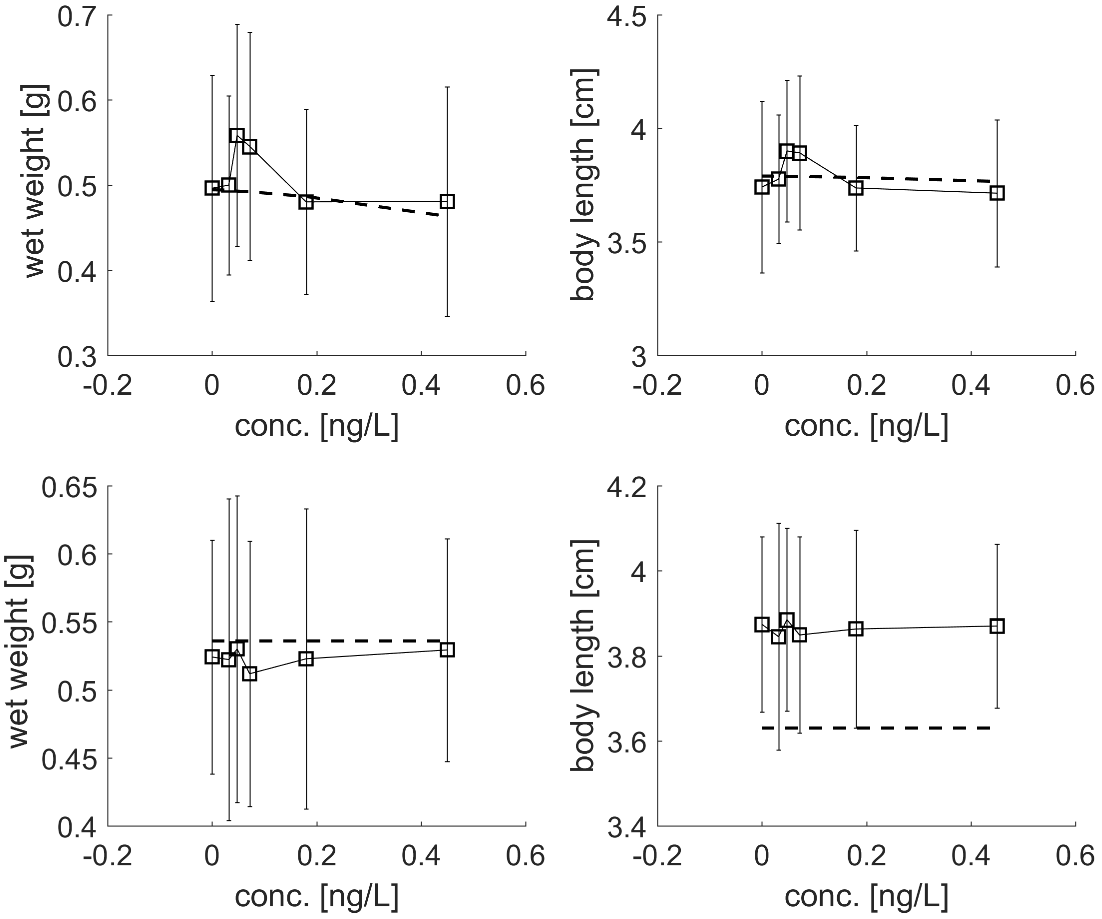

Figure S6: Results for Cohort B (upper panels) and C (lower panels) for Experiment 2. A comparison between predicted and empirical weight (left) and length (right) at test end.

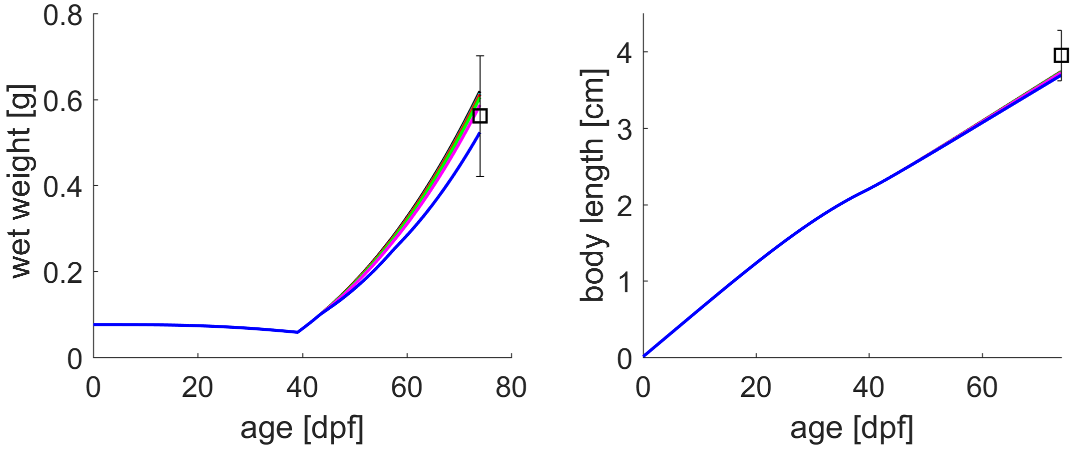

Figure S7: Experiment 2: The model predictions for cohort-A: starting with swim-ups. The predicted wet weight (left) and body length (right) are presented as a function of time. For readability, only the control data is included in this plot. The observed effect on weight is within the observed standard deviation of the control. The color coding corresponds to Figure 3 in the main manuscript.

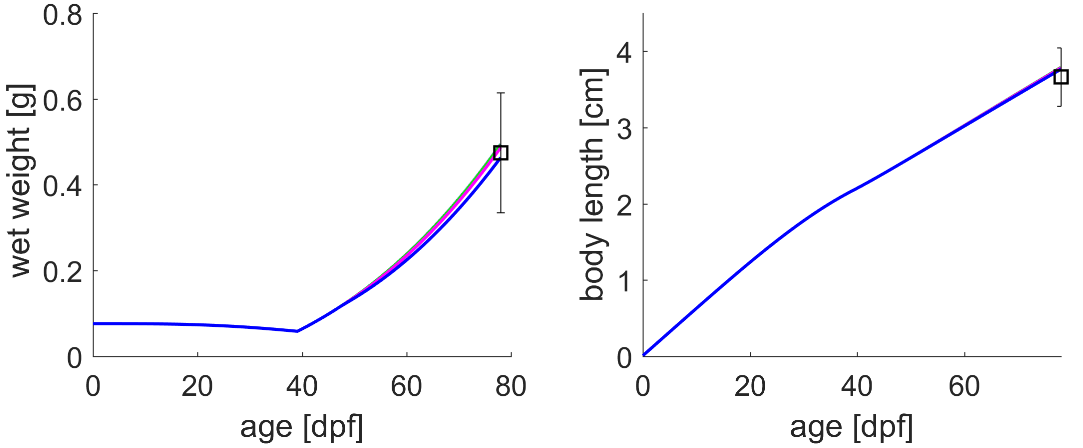

Figure S8: Experiment 2: The model predictions for cohort-B: starting with alevins. The predicted wet weight (left) and body length (right) are presented as a function of time. The observed effect on weight is within the observed standard deviation of the control, and less pronounced in comparison to cohort-A. The color coding corresponds to Figure 3 in the main manuscript.

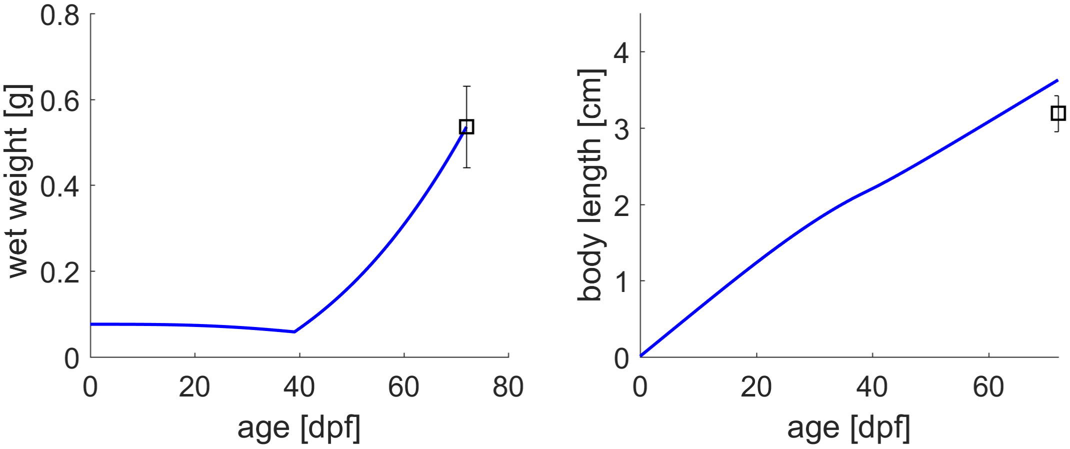

Figure S9: Experiment 2: The model predictions for cohort-C: starting with eggs. The wet weight (left) and body length (right) are presented as a function of time. For readability, only the control data is included in the plot. The fish start feeding after exposure has stopped, which is why they do not uptake the beta-cyfluthrin or exhibit any effects. The color coding corresponds to Figure 3 in the main manuscript.

1.9. Model code / user manual

Model code is available in Matlab. The code for the standard DEB model was already available from https://www.bio.vu.nl/thb/deb/deblab/add_my_pet/entries_web/Oncorhynchus_mykiss/Oncorhynchus_mykiss_res.html. Under 'Code', the Version 20170527 we used can be downloaded. In order to minimize the risk of errors in the computer code, standardized blocks of code that are prepared and verified by the curators of the Add-my-pet library (i.e. DEBtool downloaded from http://www.bio.vu.nl/thb/deb/deblab/) are used to build the model code for all Add-My-Pet entries. Each block of code is commented in a way that allows the reader to understand what is calculated. This type of documentation replaces the Pseudo-Code. We provide two different types of code: the standard Add-My-Pet-code and the simulation code. The standard Add-My-Pet-code consists of the three standard files (that can be downloaded from the Add-My-Pet website, see above) and that are setup following the templates used in the Add-My-Pet database. The Add-My-Pet-applications generally make use of the DEBtool programming toolbox, which can be downloaded from (http://www.bio.vu.nl/thb/deb/index.html). For each species, there is a "my_data file" that contains all the data (incl. references) that is used for model parametrization. The predict file holds the model equations that are used to calculate the predictions. The run file calls the necessary functions in the DEBtool programming toolbox, which then use the my_data and predict files to estimate the parameters. After downloading the species-related files and DEBtool from the web into the same folder, running the run-file in Matlab will result in print-on-screen outputs of the final parameter estimates and zero-variate data predictions (= single data points e.g. age at puberty), and popupgraphs with the data and predictions of the uni-variate data (= observations over time e.g. growth curve). More detail on how to use the Add-My-Pet-files (i.e. user manual) can be found here: http://www.debtheory.org/wiki/index.php?title=Add-my-pet_Introduction. The above mentioned files are sufficient to run the standard DEB model for rainbow trout. The code under Matlab format for calibration and validation can be provided upon request.

References

[1] Amp, online database of deb parameters, implied properties and referenced underlying data, last accessed : 2017/07/17. *Home page at: http://www.bio.vu.nl/thb/deb/deblab/add_my_pet*, 2017.

[2] S. Augustine, B. Gagnaire, M. Floriani, C. Adam-Guillermin, and S. A. L. M. Kooijman. Developmental energetics of zebrafish, Danio rerio. *Comparative Biochemistry and Physiology Part A: Molecular & Integrative Physiology*, 159(3):275–283, July 2011.

[3] P. Budy, G. P. Thiede, and T. Haddix. Rainbow trout growth and survival in flaming gorge reservoir. project xiv sport fisheries research (usu). *Annual Report*, 2002.

[4] Zhongqi Chen, Michael Snow, Craig S. Lawrence, Anthony R. Church, Shawn R. Narum, Robert H. Devlin, and Anthony P. Farrell. Selection for upper thermal tolerance in rainbow trout (Oncorhynchus mykiss Walbaum). *Journal of Experimental Biology*, 218(5):803–812, 2015.

[5] J. W. Davidson, P. B. Kenney, M. Manor, C. M. Good, G. M. Weber, A. Aussanasuwannakul, P. J. Turk, C. Welsh, and S. T. Summerfelt. Growth performance, fillet quality, and reproductive maturity of Rainbow Trout (Oncorhynchus mykiss) cultured to 5 kilograms within freshwater recirculating systems. *Journal of Aquaculture Research and Development*, 5(4), 2014.

[6] R. Froese and D. Pauly. FishBase 2017, version (june, 2017). *World Wide Web electronic publication. Home page at: http://www. fishbase. org*, 2017.

[7] Jon From and Gorm Rasmussen. Growth of rainbow trout, Oncorhynchus mykiss (Walbaum, 1792) related to egg size and temperature. *Dana*, 9:31–38, 1991.

[8] Tjalling Jager and Elke I. Zimmer. Simplified Dynamic Energy Budget model for analysing ecotoxicity data. *Ecological Modelling*, 225:74–81, January 2012.

[9] J. D. Kieffer, D. Alsop, and C. M. Wood. A respirometric analysis of fuel use during aerobic swimming at different temperatures in rainbow trout (Oncorhynchus mykiss). *Journal of Experimental Biology*, 201(22):3123–3133, November 1998.

[10] S. A. L. M. Kooijman. *Dynamic Energy Budget Theory for Metabolic Organisation*. Cambridge University Press, Cambridge, United Kingdom, 2010.

[11] Marcie M. Ninness, E. Don Stevens, and Patricia A. Wright. Removal of the chorion before hatching results in increased movement and accelerated growth in rainbow trout (Oncorhynchus mykiss) embryos. *Journal of Experimental Biology*, 209(10):1874–1882, May 2006.

[12] D. L. Straus, T. R. Stuthridge, and J. S. Anderson, S. M.and Gifford. Acute toxicity of dehydroabietic acid to rainbow trout: Manipulation of biotransformation. *Australasian Journal of Ecotoxicology*, 3:131 – 139, 1997.

[13] C. R. Tyler, T. G. Pottinger, E. Santos, J. P. Sumpter, S. A. Price, S. Brooks, and J. J. Nagler. Mechanisms controlling egg size and number in the rainbow trout, Oncorhynchus mykiss. *Biology of Reproduction*, 54(1):8–15, January 1996.

[14] F. P. J. Velsen. *Temperature and Incubation in Pacific Salmon and Rainbow Trout: Compilation of Data on Median Hatching Time, Mortality and Embryonic Staging*. Department of Fisheries and Oceans, Fisheries Research Branch, Pacific Biological Station, 1987.

[15] A. H. Weatherley and H. S. Gill. Recovery growth following periods of restricted rations and starvation in rainbow trout Salmo gairdneri Richardson. *Journal of Fish Biology*, 18(2):195–208, February 1981.

[16] Wolfgang Wieser. Developmental and metabolic constraints of the scope for activity in young rainbow trout (Salmo Gairdneri). *Journal of Experimental Biology*, 118(1):133–142, September 1985.

[17] Telat Yanik, Sükriye Aras Hisar, and Canan Bölükbasi. Early Development And Growth Of Arctic Charr (Salvelinus Alpinus) And Rainbow Trout (Oncorhynchus Mykiss) At A Low Water Temperature. 2002.

[18] Zotero. Deb library:. *https://www.zotero.org/groups/500643/deb_library/items/order/year/sort/desc*, 2018.
